# Executive Function and Epigenetic Markers in Youth Exposed to Family and Community Violence in Childhood

**DOI:** 10.1002/dev.70183

**Published:** 2026-08-02

**Authors:** Renata Queiroz Ramos, Cosme Marcelo Furtado Passos da Silva, Adriane Feijó Evangelista, Fernanda Serpeloni, Natasha Reis Lacerda, Joviana Quintes Avanci, Simone Gonçalves de Assis

**Affiliations:** ^1^ Department of Violence and Health Studies Jorge Careli National School of Public Health Sergio Arouca, Oswaldo Cruz Foundation Rio de Janeiro Rio de Janeiro Brazil; ^2^ Epidemiology in Public Health Postgraduate Program National School of Public Health Sergio Arouca, Oswaldo Cruz Foundation Rio de Janeiro Rio de Janeiro Brazil; ^3^ Department of Epidemiology and Quantitative Methods in Health National School of Public Health Sergio Arouca, Oswaldo Cruz Foundation Rio de Janeiro Rio de Janeiro Brazil; ^4^ Institute for Cancer Research Guarapuava Paraná Brazil

**Keywords:** community violence, DNA methylation, executive function, family violence, physical violence, psychological violence, youth

## Abstract

Although exposure to violence has been linked to executive functioning impairments, the biological mechanisms underlying this association remain unclear. This study examined CpG sites associated with executive functions in 78 young adults and their links to family (psychological and physical) and community violence during childhood. The scales used were Conflict Tactics (family violence); Things I Have Seen and Heard (community violence); Barkley Deficits in Executive Functioning. Mean differences (Mann–Whitney‐*U*/Kruskal–Wallis) and Spearman correlations were assessed. An epigenome‐wide association study identified differentially methylated CpGs, and linear regressions examined associations with violence exposures. Girls reported more family and psychological violence. Black participants experienced higher family violence. Executive dysfunction (ED) correlated with psychological family and community violence, and family violence forms were interrelated. Differentially methylated positions highlighted genes tied to key biological processes: *MRGPRD* (sensory neurons development); *DPPA3* (germ‐cell development); *UNKL* (cell differentiation); *GPR6* (learning and memory); *DUSP5* (cell proliferation and differentiation); and *CAPN13* (signal transduction, cytoskeletal remodeling and cell differentiation). Candidate gene analysis found associations between executive dysfunction and methylation levels in these genes. Exploratory pathway analysis suggested enrichment of neurodevelopment and synaptic plasticity, intracellular trafficking and cytoskeletal remodeling, genome stability and cellular stress responses, and neuroimmune and inflammatory signaling. These findings suggest associations between ED, childhood violence exposure, and differential DNA methylation in cognition‐ and health‐related genes, suggesting that epigenetic markers may be associated with pathways linking early adversity to later outcomes, although no causal inference can be drawn.

## 1 Introduction

Executive functions comprise a set of higher order cognitive abilities. They include higher level skills such as planning, problem‐solving, emotional regulation, and decision‐making aimed at achieving goal‐directed behavior (Alvarez and Emory 2006; Barkley 2011; Diamond 2013; Stuss and Alexander 2000; Zelazo and Carlson 2012). Core components include inhibitory control, sustained attention, cognitive flexibility, and working memory (Baddeley 2010; Cordeiro and Minervino 2024; Dias 2019). These domains are modulated by different neurochemical systems, such as dopamine and noradrenaline (Arnsten 2009), and involve several brain structures (Aston‐Jones and Cohen 2005).

From a developmental perspective, executive functions emerge from early attentional and regulatory systems and progressively differentiate as prefrontal–parietal networks mature. In early childhood, executive functioning is expressed through basic inhibitory control, working memory, and cognitive flexibility, highly scaffolded by caregivers. During middle childhood, these skills integrate to support rule‐guided behavior and academic functioning. In adolescence, ongoing prefrontal maturation enables more complex planning but increases context sensitivity. Childhood and adolescence are critical stages for neurological, behavioral, and social development, marked by heightened neuroplasticity (Bronfenbrenner 1977; Martins and Szymanski 2004), which directly affect the consolidation of executive functioning. In adulthood, executive functioning reaches peak efficiency before gradual age‐related decline (Diamond 2013; Luna et al. 2015). Executive dysfunctions (EDs) may persist and manifest differently throughout the life course, with distinct behavioral profiles observed during childhood, adolescence, and adulthood (Correia 2013; Margari et al. 2016).

Adverse childhood and adolescent experiences, such as violence, are strongly associated with cognitive impairments, including executive functions (Ainamani et al. 2017; Bogliacino et al. 2017; Passos 2019; Romero‐Martínez et al. 2021). Deficits in executive functions may manifest as difficulties in emotional regulation, impulsivity, disorganization, and attentional problems (Margari et al. 2016; Roberts and Pennington 1996). Acute stress may transiently enhance executive alertness, characterized by increased executive control and attentional focus that facilitate rapid, goal‐directed responses (Weinbach and Henik 2012). However, chronic or unpredictable stress is linked to long‐term impairments in working memory and neuroplasticity (Yuen et al. 2017).

Relating to theories about exposure to violence, ecological model proposed by the World Health Organization (Dahlberg and Krug 2006) presents a complex and multifactorial phenomenon resulting from the interaction of factors operating at individual, relational, community, and societal levels. Within this framework, interpersonal violence occurs between individuals and is classified into family or intimate partner violence (typically taking place within the home) and community violence, which occurs between individuals who may or may not know each other, generally outside the domestic setting. family violence includes child abuse, if perpetrated by parents or caregivers and includes, among others, physical aggression (e.g., hitting or kicking) and psychological abuse (e.g., threats, humiliation, and coercive control), and constitutes a major determinant of injury, mental disorders, and adverse health outcomes across the life course (Glaser 2002; Straus et al. 1998; Teicher and Samson 2016; World Health Organization 2002, 2016). Evidence indicates that children and adolescents are highly exposed to different forms of violences, as victims or witnesses of events such as shootings, physical and verbal assaults, and exposure to injured or deceased individuals (Pinto and Assis 2013). Beyond these contexts, additional environmental risk factors, such as poverty and social marginalization (Haushofer and Fehr 2014; Mani et al. 2013), compound these effects, contributing to long‐term consequences for cognitive development and emotional regulation (McLaughlin et al. 2014). The experience of violence is strongly associated with cognitive and mental health impairments, particularly by its frequency, severity, cumulative, and when exposure occurs in sensitive developmental periods. The cumulative exposure, known as polyvictimization, refers to the co‐occurrence of multiple types of victimization and has been identified as a potential predictor of psychopathology and developmental disruption (Finkelhor et al. 2007; Wolfe 2018). Research has shown that poly‐victimized youth are at significantly higher risk for anxiety, depression, PTSD, and deficits in executive functions compared to those who experience single forms of adversity (Turner et al. 2010).

Studies with different methodologies, including large samples and meta‐analysis, indicate that multiple executive domains are affected by experiencing violence and that the effects persist into adulthood and are moderated by the type and timing of adversities (Clinchard et al. 2024; Letkiewicz et al. 2021; Op Den Kelder et al. 2018). Other empirical studies have demonstrated similar effects, including deficits in attention and memory tasks in maltreated institutionalized children between 10 and 15 years old (Correia 2013) and in medication‐naive children (5–12 years of age) with a history of early severe trauma from a foster care home (Bücker et al. 2012); attentional and cognitive flexibility impairments in children (7–12 years) in a psychiatry institution, victims of sexual abuse (Marques 2015); working memory and psychosocial functioning impairments in people (age range from 18 to 65) in a Refugee Settlement who experienced previous violence (Ainamani et al. 2017). Colombian civilians (18 and 24 years of age) who were exposed either to urban violence or to warfare more than a decade earlier showed reduced short‐term memory and executive performance, suggesting a long‐term effect of unresolved trauma (Bogliacino et al. 2017).

The dimensional model of adversity and psychopathology (DMAP) organizes early‐life adversities along two distinct dimensions of environmental experience—threat and deprivation—each proposed to influence psychopathology through separable neurodevelopmental pathways (McLaughlin et al. 2014; Larkin and Shelleby 2026). Threat dimensions, encompassing experiences involving harm and the threat of harm, are theorized to disrupt neural systems related to the amygdala and prefrontal circuitry, leading to difficulties in emotional regulation and heightened vulnerability to attention, internalizing and externalizing disorders. Deprivation dimensions, encompassing neglect and limited cognitive and social stimulation, are theorized to impair cognitive development, especially executive functioning and language, by altering cortical development and synaptic pruning, thereby increasing risk for psychopathology through distinct mechanisms (Larkin and Shelleby 2026). The model further emphasizes that the developmental timing of exposure, cumulative burden, and contextual moderators (e.g., caregiving quality and social environment) shape heterogeneity in outcomes, such that individuals exposed to similar adversities may follow different trajectories depending on when and how those experiences occur (McLaughlin et al. 2014; Larkin and Shelleby 2026).

In parallel with developmental models, violence and other adverse events also exert an impact on *Epigenetics*, which refers to a set of biological mechanisms through which gene expression is regulated without altering the underlying DNA sequence. One of the most studied mechanisms is DNA methylation, which involves the addition of a methyl group (CH_3_) to the fifth carbon of cytosine, primarily at CpG dinucleotides, catalyzed by DNA methyltransferases (Franklin et al. 2012). These modifications can silence or activate genes and are sensitive to environmental stimuli, thus acting as a molecular memory of life experiences (Champagne 2010; Champagne and Curley 2011; Weaver et al. 2004). Epigenetics has become increasingly relevant in public health. Although in cancer research, epigenetics is advancing diagnostics, prognostics, and therapeutic responses by identifying key biomarkers, their application in mental health, for example, remains more recent and methodologically challenging. Mental disorders lack clear biomarkers and involve complex interactions among neurotransmission, neuroplasticity, and stress–response pathways across various brain regions (Schiele and Domschke 2018).

Emerging research in epigenetics highlights the long‐term impact of trauma and adversity across the lifespan, showing that such experiences can induce stable epigenetic modifications. For instance, intimate partner violence during pregnancy has been linked to increased methylation of the glucocorticoid receptor (GR) gene in offspring (Radtke et al. 2011), and stress‐related epigenetic marks have been shown to extend across three generations (Serpeloni et al. 2017). Violent experiences and other environmental stressors can influence key biological systems, including circulatory (Dos Santos Oliveira et al. 2023), immune (Chen et al. 2021), and endocrine pathways (Wen et al. 2024), and may accelerate biological aging via epigenetic clocks (dos Santos Oliveira et al. 2023). Moreover, both interpersonal and social violence are associated with epigenetic alterations (Shields 2017), psychiatric conditions (e.g., PTSD, anxiety, and depression), and cognitive impairments such as ED (Serpeloni et al. 2020).

Despite growing evidence linking violence exposure, executive functioning, and epigenetic mechanisms, studies integrating these domains within a developmental framework remain limited. To address this gap, this article aims to investigate associations between DNA methylation (epigenome‐wide association study [EWAS]), candidate genes (theory‐driven and based on EWAS results), and executive functions in youth, considering family and community throughout childhood development in a cohort monitored between 2005 and 2022. Grounded in this developmental and biological framework, we hypothesize that (i) ED would be associated with differential DNA methylation at specific CpG sites; and (ii) there would be association between methylation signals with executive dysfunction and violence exposure.

## 2 Materials and Methods

### 2.1 Participants

This study draws on data from a longitudinal cohort in São Gonçalo, Rio de Janeiro, one of the state's most populous cities, with 896,744 residents as of 2022 (Brazilian Institute of Geography and Statistics [IBGE] 2022). The cohort has been a key resource for investigating childhood development in contexts of socioeconomic adversity, mental health problems, community, and domestic violence (Pinto and Assis 2013; Assis et al. 2011; Avanci et al. 2012; Pires et al. 2013; Silva Filho et al. 2023). All stages of this study were approved by the Ethics Committee of the Oswaldo Cruz Foundation (CAAE 18723119.0.0000.5240); all participants signed declarations of free and informed consent.

Data collection occurred at five time points: 2005 (baseline), 2006 (1‐year), 2008 (3‐year), 2012/2013 (8‐year), and 2021/2022 (16‐year follow‐up). The initial sample comprised 500 first graders; by the final wave, 129 remained. Participants had a mean age of 7.6 years (SD = 0.9) in 2005 and 23.5 years (SD = 1.1) in 2022. Questionnaires were completed by mothers or guardians in the first three waves and by the youth themselves in the third, fourth, and fifth waves. Consistency across waves was ensured by using the same instruments and standardized data collection procedures, implemented by the same research team, thereby minimizing heterogeneity and supporting comparability over time. This longitudinal study was designed to assess childhood development across multiple time points; however, data collection was dependent on funding and operational resources, resulting in some intervals without assessments.

In 2021/2022, saliva samples were collected from 112 participants who consented to the epigenetic analysis, out of the 129 young people located in that wave. After quality control, 107 samples were retained. Only participants with complete data on exposure to violence across all five waves were included, yielding a final sample of 78 individuals. Sociodemographic changes were observed, indicating that the reduced sample may differ slightly from the full cohort interviewed in the last cohort's wave (Table S1).

### 2.2 Measures

#### 2.2.1 Family Violence (FamV—161 Items)

Variables were derived from two scales administered at each assessment. The Conflict Tactics Scale—CTS, Child Form R (Hasselmann and Reichenheim 2003; Straus 1979; Straus and Gelles 1995) measures physical and psychological family violence from parents toward children. The research was conceived prior to the Portuguese validation of the Conflict Tactic Scales Parent Child (CTSPC); it is still used in longitudinal studies (Yaros et al. 2016; Simons and Wurtele 2010). Although the CTS records event frequency, responses were dichotomized here as “yes” (≥1 event) or “no” (0 events). This construct showed a score range from 67.9 to 108.4. The CTS is cross‐culturally validated, with its physical aggression subscale demonstrating good reliability (Cronbach's *α* > 0.70) (Hasselmann and Reichenheim 2003). Witnessing parental verbal aggression (2008, 2012, and 2022) was assessed using an item from the Things I Have Seen and Heard (TISH) scale (“Have you heard your parents yelling at each other?”) (Malik 2008; Richters and Martinez 1993). The Portuguese version showed strong reliability (Cronbach's *α* = 0.763), and responses were similarly dichotomized. The internal consistency in our sample varied across waves, with acceptable to good reliability in 2006 (*α* = 0.81), 2008 (*α* = 0.81), and 2012 (*α* = 0.88), but lower values in 2005 (*α* = 0.53) and 2022 (*α* = 0.50). These fluctuations likely reflect differences in sample characteristics, item variability, or changes in response patterns over time. Overall, the results suggest that the scale performed adequately in most waves, whereas lower reliability in specific years should be interpreted with caution. The family violence construct was divided into physical (92 items) and psychological (69 items) dimensions spanning 2005–2022. Analyses examined overall family violence and its subtypes, focusing solely on the child as victim or witness.

#### 2.2.2 Psychological Family Violence (PsyFV—69 Items)

It includes insults, humiliation, threats, object throwing, and witnessed verbal aggression from parents toward the child (Hasselmann and Reichenheim 2003; Straus 1979), supplemented by a TISH item already mentioned. Caregivers completed 52 items (2005–2008); youth answered 17 (2012–2022). This construct showed a score range from 31.8 to 59.4.

#### 2.2.3 Physical Family Violence (PhyFV—92 Items)

It assesses minor and severe physical aggression by fathers, mothers, or guardians, such as pushing, slapping, hitting, kicking, burning, or threats/use of weapons (Hasselmann and Reichenheim 2003; Straus 1979). Caregivers answered 72 items (2005–2008), and participants completed 20 items (2012–2022), with behaviors reported separately for mothers and fathers. This construct showed a score range from 0 to 34.0.

#### 2.2.4 Community Violence (ComV—51 Items)

In 2005–2006, caregivers reported exposure to events like serious injuries, unsafe environments, home robberies, and community shootings. From 2008 onward, youth completed the TISH scale (Malik 2008), covering experiences such as beatings, shootings, gunfire, and arrests. Responses ranged from 0 (“never”) to 4 (“often”). A total of 15 items were completed by caregivers (2005–2006) and 36 by youth (2008–2022). In a comparable sample, the scale showed good reliability (Cronbach's *α* = 0.763) (Ximenes et al. 2013). The Cronbach *α* in our sample was generally acceptable to good, particularly in 2008 (*α* = 0.77), 2012 (*α* = 0.82), and 2022 (*α* = 0.84), indicating more stable internal consistency across waves. Although a lower value was observed in 2006 (*α* = 0.54), the overall pattern supports the reliability of the measure, suggesting that the community violence construct was more consistently captured across time. This construct showed a score range from 2.0 to 34.5.

Violence scores were calculated by summing the frequencies of all items and adjusting the total by multiplying it by the ratio between the total number of items each year and the number of valid (non‐missing) responses provided by each participant. This approach accounts for missing data while preserving comparability across individuals. The final scores were then categorized into two exposure groups: high (top tertile) and low (remaining participants). We categorized violence variables to enhance the interpretability of comparisons between high‐ and lower risk groups, considering the high prevalence of exposure in the study population.

#### 2.2.5 Executive Dysfunction (ED) (2022)

The Barkley Deficits in Executive Functioning Scale (BDEFS) short form (20 items) measures future‐oriented self‐regulation across five domains: time management, organization/problem solving, self‐restraint, self‐motivation, and emotion regulation. Items are scored from 0 (“rarely/never”) to 4 (“very often”) and summed for a total score. Scores are interpreted using age‐based percentiles (normal: <44 for ages 21–24; <42 for ≥25), with no sex differences. The Brazilian version demonstrated high reliability (*α* = 0.911) (Barkley 2011; Godoy et al. 2015, 2018). This scale showed a score range from 20.0 to 74.0.

#### 2.2.6 Saliva (2022)

Saliva samples were collected on the same day participants completed the questionnaire, using a non‐invasive Oragene‐Discover (OGR‐500) Collection Kit (DNA Genotek, Ontario, Canada). To ensure sample quality, participants drank a glass of water and then abstained from food and water for 30 min prior to collection. Of the 129 participants, 17 did not provide samples due to refusal, absence of collection kits, or completing the survey remotely. Thus, 112 samples were used for epigenetic and epigenomic analysis (EWAS), assessing whole‐genome DNA methylation at ∼850,000 CpG sites (Infinium Human Methylation EPIC BeadChip).

Samples were randomized to reduce bias from sex, age, or lab conditions, and all procedures were conducted in a single time window to control for environmental variation. Processing was performed at the Life & Brain facility (University of Bonn, Germany). Preprocessing (*N* = 112) was carried out using the ChAMP package (Morris et al. 2014; Tian et al. 2017) in R (v4.2.2), which evaluated hybridization signal quality, bisulfite conversion efficiency, and negative controls. Batch correction addressed technical variability, and Beta Mixture Quantile (BMIQ) normalization corrected probe‐type bias. Beta values ranged from 0 (unmethylated) to 1 (fully methylated). Data were organized into a multi‐assay experiment (MAE) format in R/Bioconductor. Five samples were excluded for failing quality control, resulting in a final dataset of 107 samples. Candidate genes were chosen on the basis of theory‐driven correlations between executive function and violence, in addition to those that stood out in the EWAS results. Analyses of each gene were performed separately for executive function and violence exposure.

### 2.3 Statistical Analysis

#### 2.3.1 Frequency Measures

Descriptive analyses were conducted, including frequency counts for categorical variables, median (Md) and interquartile ranges (IQRs) for continuous variables. Due to the non‐normal distribution of the variables, non‐parametric statistical tests were applied: the Mann–Whitney *U* test for comparisons between two groups; the Kruskal–Wallis test for comparisons among three or more groups, and Spearman's rank correlation for assessing associations between continuous variables.

#### 2.3.2 Epigenome‐Wide Association Study (EWAS)

Beta values were transformed into log 2 ratios (*M* values), as they offer improved performance in terms of detection rate (DR) and true positive rate (TPR) due to their closer approximation to homoscedasticity (Du et al. 2010). Sex and epithelial cell‐type fraction were included as covariates in all models. Cell‐type composition was estimated using a reference‐based deconvolution approach implemented in EpiDISH. Estimated epithelial cell proportions were subsequently incorporated as covariates in sensitivity EWAS models fitted with *limma*. Previous research has demonstrated that saliva represents a heterogeneous mixture of epithelial and immune cells, and variation in cell composition may influence DNA methylation profiles (Middleton et al. 2022). Therefore, additional analyses adjusting for estimated epithelial cell fractions were performed to evaluate the robustness of the observed associations to potential cellular heterogeneity. Because the estimated epithelial and non‐epithelial cell fractions are complementary, only the epithelial cell fraction was included as a covariate in the adjusted models to avoid collinearity (Chan et al. 2026).

The *rafalib* package was used to assess the effect size of methylation of EF compared to ED, represented by a volcano plot (Irizarry and Love 2025)—CpG sites annotated to candidate genes previously implicated in executive functioning and exposure to violence were highlighted to facilitate interpretation (Lacerda et al. 2025). The *limma* package (Ritchie et al. 2015) was used to assess CpGs overlap—represented with the *VennDiagram* package—and the differentially methylated positions (DMPs)—representation with the *qqman* package and tables. Study‐design sampling weights were incorporated into all EWAS models. The Benjamini–Hochberg false discovery rate (FDR) correction for multiple‐testing was applied and both adjusted and unadjusted results were reported. In addition, a suggestive significance threshold based on raw *p* values (*p* = 1 × 10^−^^5^) was used to highlight loci that did not pass FDR correction but may warrant further investigation. The gene names and characteristics of CpGs were accessed in Infinium MethylationEPIC v1.0 B5 Manifest File (Illumina 2020), and its functions in NCBI Gene (National Center for Biotechnology Information [NCBI] n. d.), Stelzer et al. (2016) and Ensembl (Cunningham et al. 2022; Harrison et al. 2024).

#### 2.3.3 Candidate Genes

Linear regression models were used to assess the associations between DNA methylation Beta values and ED, family violence, psychological family violence, physical family violence, and community violence. All models were adjusted by sex and epithelial cell composition. Study‐design sampling weights were incorporated into all models. Graphical representations were generated using the *ggplot2* package in R.

DNA methylation was assessed at follow‐up, and findings should, therefore, be interpreted as associations rather than causal effects. To minimize multicollinearity, highly correlated violence domains were not included simultaneously; models instead used either FamV with ComV or PsyFV and PhyFV with ComV, and correlations suggested minimal impact on the results. A significant level of 5% was adopted for all analyses. All statistical analyses were conducted using R software (version 4.1.1; R Foundation for Statistical Computing, Vienna, Austria) and RStudio (version 2024.04.2 + 764). To enhance the biological interpretation of the EWAS findings, we performed an exploratory pathway enrichment analysis using the Reactome database, implemented in R with the ReactomePA package. Genes identified from the EWAS results, and candidate gene analyses were used as input for an overrepresentation analysis to explore potential enrichment of biological pathways related to ED and exposure to violence.

## 3 Results

The descriptive and analytical results are detailed in the following sections. To facilitate understanding, details about the subtypes of family violence (physical and psychological) were moved to the Supporting Information section.

### 3.1 Sociodemographic Characteristics

We examined the sociodemographic profile of the participants (*N* = 78), including sex, age, skin color, and socioeconomic stratum (Table 1). The median scores of family violence (FamV) between 2005 and 2022 were higher among girls (Md = 87.9) compared to boys (Md = 81.9) (Mann–Whitney‐*U W* = 953, *p* = 0.045), so as the psychological family violence (PsyFV) in girls (Md = 45.9) compared to boys (Md = 41.8) (Mann–Whitney‐*U W* = 979, *p* = 0.023). Regarding skin color, higher median scores of FamV were obtained for black (Md = 90.0) and brown (Md = 83.0) participants (Kruskal–Wallis *χ* ^2^ = 12.075, *p* = 0.002). Similarly, black participants presented higher scores of PsyFV (Md = 47.4), as did brown individuals (Md = 42.4) (Kruskal–Wallis *χ* ^2^ = 11.727, *p* = 0.003). Physical family violence (PhyFV) was much lower in white individuals compared to the other skin color categories. No statistically significant differences were found regarding age or socioeconomic status.

**TABLE 1:** Median, interquartile range (IQR), and statistical tests for median differences in violences and executive dysfunction according to socioeconomic characteristics (*N* = 78).

Variables	Family violence (2005–2022)^1^			Psychological family violence (2005–2022)^2^			Physical family violence (2005–2022)^3^			Community violence (2005–2022)^4^			Executive dysfunction (2022)^5^		
	Median	IQR	*p* value	Median	IQR	*p* value	Median	IQR	*p* value	Median	IQR	*p* value	Median	IQR	*p* value
**Sex** (*N* = 78)															
**Female** (*N* = 43, 55.1%) **Male** (*N* = 35, 44.9%)	87.9 81.9	12.5 9.5	**0.045^8^**	45.9 41.8	10.2 6.1	**0.023^8^**	7.0 7.0	5.5 8.0	0.860^8^	12.0 14.0	6.3 6.2	0.251^8^	30.0 27.0	15.5 13.5	0.113^8^
**Age** (*N* = 78)															
**22–24** (*N* = 70, 89.7%) **25+** (*N* = 8, 10.3%)	82.9 84.3	13.8 9.1	0.979^8^	42.9 43.6	9.7 5.8	0.930^8^	7.86 6.0	6.7 4.6	0.386^8^	13.0 10.0	7.9 5.4	0.195^8^	30.0 29.0	14.5 11.0	0.786^8^
**Skin color^6^** (*N* = 78)															
**White** (*N* = 20, 26.3%) **Black** (*N* = 26, 34.2%) **Brown** (*N* = 32, 39.5%)	78.5 90.0 83.0	7.0 13.9 8.5	**0.002^9^**	40.4 47.4 42.4	7.1 8.7 7.7	**0.003^9^**	4.0 8.6 8.0	4.5 7.1 6.1	**0.019^9^**	10.8 13.1 13.0	8.1 5.0 8.0	0.066^9^	30.0 30.0 27.5	10.2 17.5 15.0	0.477^9^
**Socioeconomic stratum (SES)^7^** (*N* = 63)															
**Upper** (*N* = 11, 17.5%) **Middle/Low** (*N* = 52, 82.5%)	80.9 84.3	13.1 12.9	0.581^8^	42.9 43.9	9.3 8.7	0.717^8^	8.0 6.5	5.0 7.5	0.807^8^	11.0 12.0	7.0 7.5	0.657^8^	38.0 28.5	19 15.2	0.866^8^

*Note:* *P*‐values < 0.05 are highlighted in bold; ^1^Range from 67.9 to 108.4; ^2^ range from 31.8 to 59.4; ^3^ range from 0 to 34.0; ^4^ range from 2 to 34.5; ^5^ range from 20 to 74; ^6^ The study had one Indigenous/Asian participant that was incorporated into the Brown category, close to the term “Pardo” in Brazil, used to describe people of mixed racial backgrounds, especially those with Indigenous, African, and European ancestry; ^7^ calculated by educational level of parents and household items, Brazil's Criteria/ABEP; ^8^ Mann–Whitney‐*U* Test; ^9^ Kruskal–Wallis Test.

### 3.2 ED and Violence: Correlation Between Variables

The variable ED was correlated with psychological family violence (*r*_s_ = 0.31, *p* = 0.007) and community violence (*r*_s_ = 0.23, *p* = 0.04) (see Figure S1). FamV was correlated to PsyFV (*r*_s_ = 0.94, *p *< 0.001) and PhyFV (*r*_s_ = 0.76, *p* < 0.001); PsyFV and PhyFV were also correlated (*r*_s_ = 0.62, *p* < 0.001), pointing to an intricate proximity between the two forms that make up family violence. FamV and ED were not correlated (*r*_s_ = 0.22 *p* = 0.052, data not shown).

### 3.3 Exploratory Analysis of Epigenome

**FIGURE 1:**
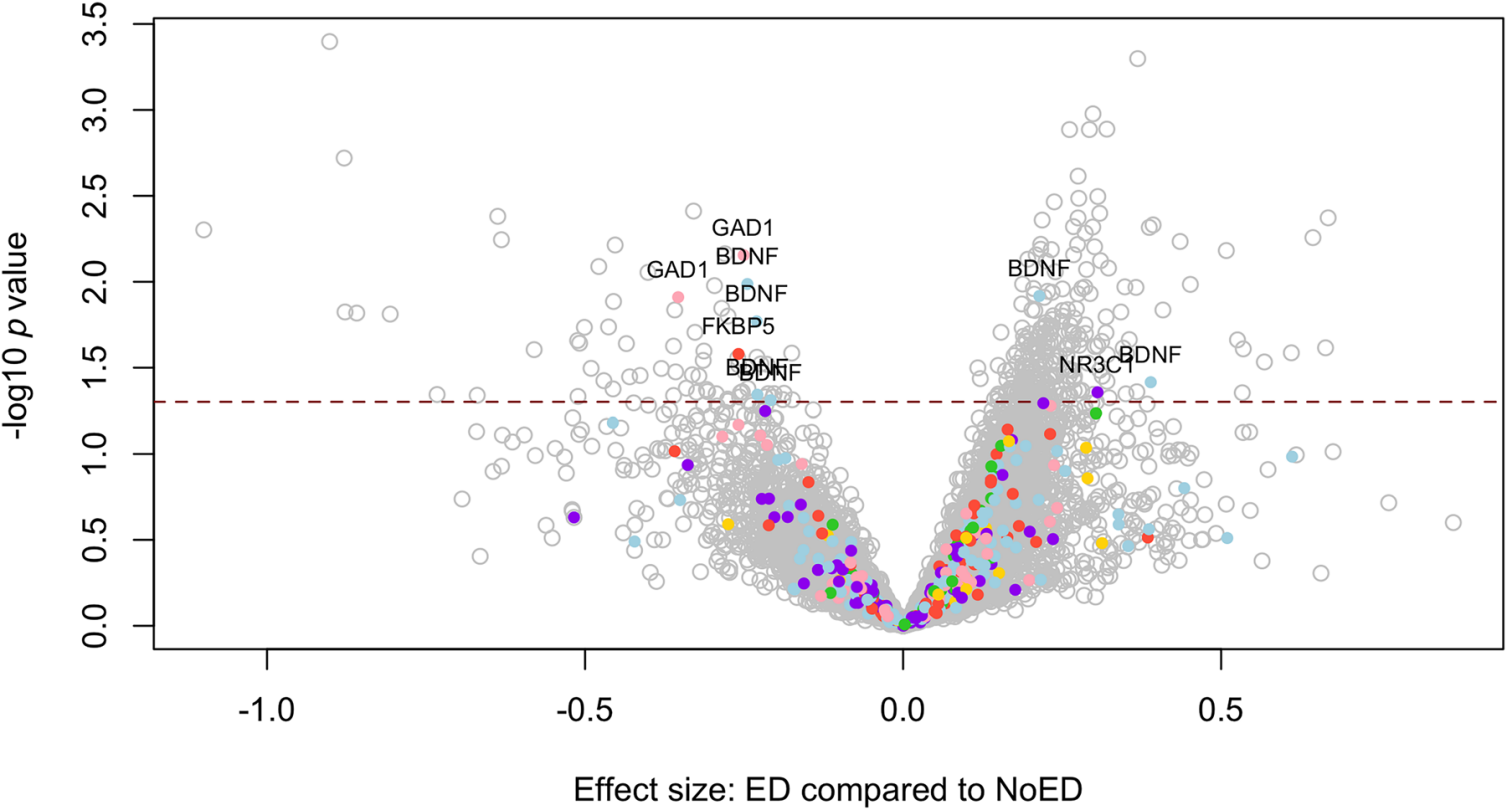
Volcano plot of executive dysfunction (ED) CpGs distribution (*M* values) compared to individuals with no executive dysfunction (NoED), adjusted by sex and epithelial cell composition. The horizontal dashed line indicates the nominal significance threshold (*p* = 0.05); there were no significant CpGs when the Benjamini–Hochberg (BH) 5% FDR correction (*p* = 5 × 10^−7^; −log _10_(*p*) ≈ 6.3) was used. All CpGs are shown in grey, whereas CpGs annotated to candidate genes are highlighted in distinct colors: *BDNF* (blue), *SLC6A4* (green), *NR3C1* (purple), *FKBP5* (red), *OXTR* (yellow), and *GAD1* (pink). Gene labels correspond to the most significant CpG per gene among those surpassing the significance threshold. CpG sites corresponding to candidate genes previously implicated in executive functioning and exposure to violence were highlighted to improve interpretation of the results (Lacerda et al. 2025).

First, we performed an exploratory analysis of the variable ED (Figure 1). The red dashed line represents the nominal *p* value threshold (*p* = 0.05, −log _10_ *p* ≈ 1.3). A moderate number of CpG sites exceeded this threshold, with the majority showing a positive effect size. This suggests that individuals with ED symptoms could exhibit more hypermethylated CpG sites compared to those without ED symptoms.

In the Supporting Information section, one can assess the overlap of CpG sites associated with ED and family/community violence, using a cutoff of the 10,000 most significant CpGs (Figure S2). The nine genes identified in this EWAS clustered into three interconnected biological domains: regulation of gene expression and genomic stability (*SUPT5H*, *HLTF*, *RBPMS*, and *HRCT1*), cellular architecture and intracellular homeostasis (*MSTO1*, *CDK5RAP2*, *CFAP61*, and *AGAP3*), and neurodevelopmental and synaptic processes (*AGAP3*, *RBPMS*, and *CDK5RAP2*). Collectively, these pathways are involved in transcriptional regulation, DNA repair, cytoskeletal organization, intracellular trafficking, neuronal differentiation, and neural plasticity, highlighting mechanisms relevant to brain development and cognitive functioning. The cg08745216 is not annotated. There is also an overlap of ED broken down by psychological violence (PsyFV) and physical violence (PhyFV), and community violence (ComV) pointed out in Figure S3. Only one CpG overlapped between all variables when including physical and psychological violence in the family: cg01619156, not annotated.

### 3.4 Differentially Methylated Positions

**FIGURE 2:**
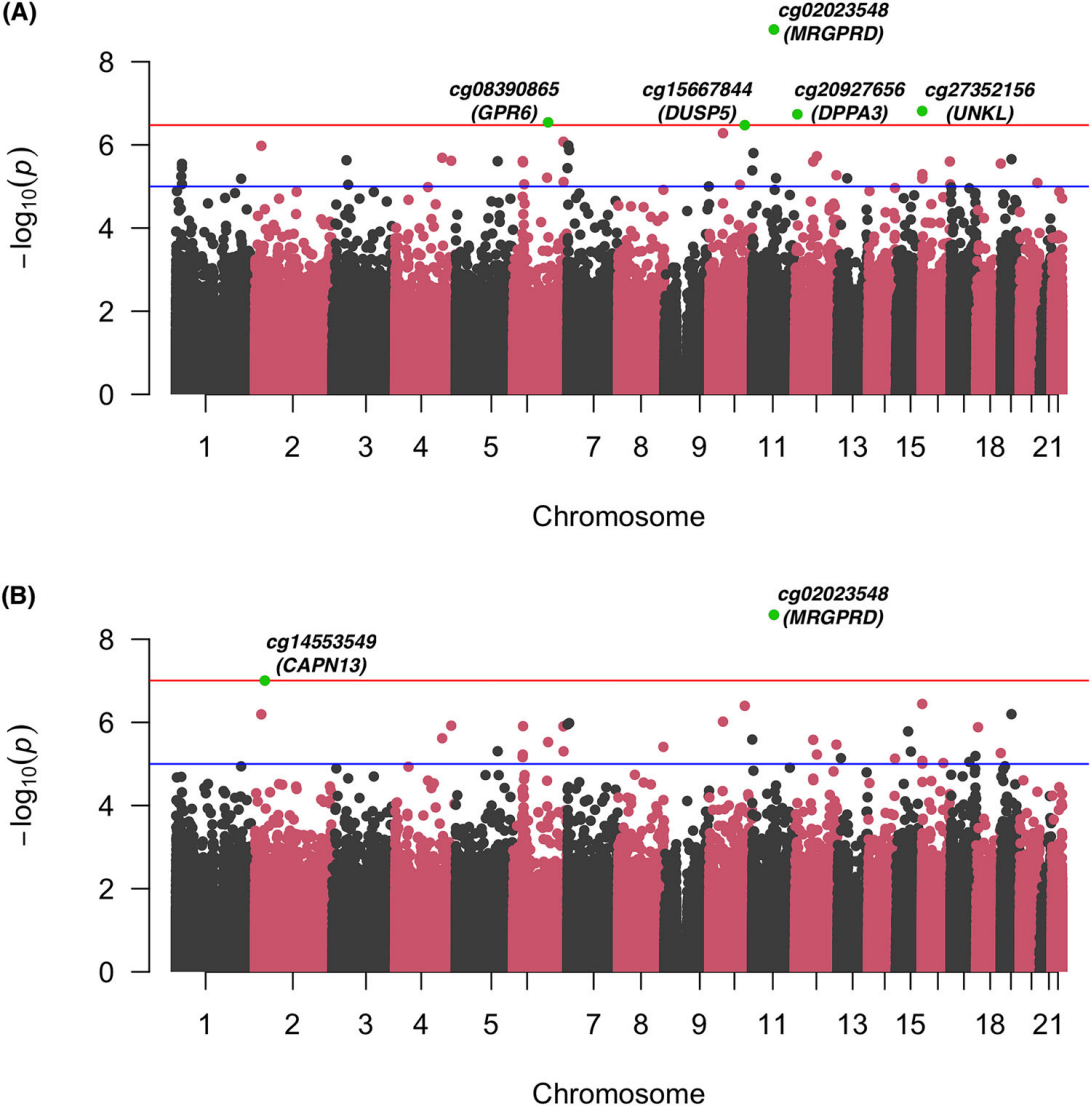
Manhattan plot showing significant CpG sites from the executive dysfunctions (ED) model and types of violence: (A) FamV and ComV; and (B) PsyFV, PhyFV, and ComV, both adjusted by sex and epithelial cell composition. The red line represents the multiple testing significance threshold, corresponding to the raw *p* value at which results pass the Benjamini–Hochberg (BH) 5% FDR correction (*p* = 5 × 10^−7^; −log _10_(*p*) ≈ 6.3). The blue line represents a suggestive significance threshold based on raw *p* values (*p* = 1×10^−5^; −log _10_(*p*) ≈ 5.0), less stringent than the Benjamini–Hochberg (BH) 5% FDR correction.

To evaluate the distribution of differentially methylated CpG sites, we generated a Manhattan plot for ED related to FamV and ComV (Model 1) and PsyFV, PhyFV, and ComV (Model 2), both adjusted by sex and epithelial cell composition (Figure 2). The red line represents the multiple testing significance threshold (Benjamini–Hochberg method), with 5 CpG sites surpassing this cutoff (Figure 2A): The cg02023548 of the *MRGPRD* gene (Mas related GPR family member D) stands out, being involved in the signaling pathway of the receptor related to angiotensin‐mediated vasodilation (log FC = 0.402, *p* = 0.001) (Meixiong and Dong 2017). The cg20927656, corresponding to *DPPA3* gene (developmental pluripotency‐associated protein 3), is involved in pluripotency maintenance and has already been linked to germ‐cell development, early embryonic reprograming and epigenetic reprograming (log FC = −1.219, *p* = 0.045) (Toriyama et al. 2024). The cg27352156 (*UNKL*, chromosome 16—unk like zinc finger) (log FC = 0.404, *p* = 0.045) is hypermethylated, and it is responsible for encoding a RING finger protein that may play a role in Rac signaling. It binds to Brg/Brm‐associated factor 60b (BAF60b) and can promote its ubiquitination involved in cellular stress responses. The cg08390865, gene *GPR6* (G protein‐coupled receptor 6), is possibly involved in in pathways related to neuronal signaling, synaptic transmission, and neurodevelopment (log FC = −0.520, *p* = 0.049) (Barekatain et al. 2024). Lastly, the cg15667844, related to the *DUSP5* (dual specificity phosphatase 5), is associated with cell proliferation and differentiation and cellular responses to environmental stimuli (log FC = 0.597, *p* = 0.049) (Lake et al. 2016). At the less stringent raw *p* value threshold (blue line), 43 CpG sites were identified.

In Model 2 (Figure 2B), *MRGPRD*, already seen in Figure 2, remains significant (cg02023548, log FC = 0.392, *p* = 0.002). Also significant, the cg14553549 of the *CAPN13* gene, has already been linked to regulated proteolysis, cytoskeletal remodeling, signal transduction, protein turnover, and cellular stress responses (log FC = 0.447, *p* = 0.036) (Metwally et al. 2023). At the less stringent raw *p* value threshold (blue line), 34 CpG sites were identified.

We conducted models to assess the association between ED and various types and subtypes of violence. Table S2 presents the top 50 CpGs/genes associated with FamV + ComV and Table S3 discriminates types of family violence. The gene repeated between models is *MRGPRD*, already signed, that encodes a G‐protein‐coupled receptor involved in sensory neuronal signaling, vascular regulation, and neuroimmune communication, acting as central to peripheral pain, chronic itch, and suppressing inflammation.

### 3.5 Candidate Genes

The CpGs/genes identified in the combined models (ED with [FamV + ComV]) and (ED with [PsyFV + PhyFV + ComV]), both adjusted by sex and epithelial cell composition, were further examined in individual linear regression models: *UNKL* (cg00329656), *MRGPRD* (cg02023548), *GPR6* (cg08390865), *DUSP5* (cg15667844), *DPPA3* (cg20927656), and CAPN13 (cg14553549) in Figures S4–S9. ED was significantly associated with methylation levels in several loci: *MRGPRD* (*β* = 0.0009, *p* = 0.00019); *UNKL* (*β* = 0.0006, *p* = 0.00057); *DPPA3* (*β* = −0.0041, *p* = 0.021); *DUSP5* (*β* = −0.0018, *p* = 0.016); *GPR6* (*β* = −0.0003, *p* = 0.0000014); *CAPN13* (*β* = 0.0003, *p* = 0.039).

In addition to these data‐driven findings, we also evaluated CpGs in genes previously implicated in ED, on the basis of a targeted literature review carried out by our research group (Lacerda et al. 2025), obtaining the following results (Figures S10 and S11): *NR3C1* (cg08845721, *β* = 0.0012, *p* = 0.032) and *FKBP5* (cg10300814, *β* = 0.0008, *p* = 0.028) were associated to PhyFV, and *NR3C1* was also associated to ED (*β* = −0.0007, *p* = 0.022). *BDNF* (cg01225698), 5‐HTT/*SLC6A4* (cg12074493), *OXTR* (cg00385883) and *GAD1* (cg19374317) were also investigated but did not indicate methylation changes related to ED or violences.

To provide additional biological context to the identified genes, an exploratory Reactome pathway analysis with Top 50 CpGs was performed (Tables S2 and S3). Model 1 (Table S2) functional annotation revealed convergence across several interconnected biological pathways. A substantial proportion of genes was involved in neuronal development, synaptic signaling, and neural plasticity, including *GPR6, SYNJ2, LRP1, GPM6A, GDPD5*, and *AKT1*. A second cluster comprised genes related to genome maintenance and DNA damage responses, such as *HLTF, FAAP24, TELO2, EME2*, and *MDM2*. Additional genes participated in transcriptional and posttranscriptional regulation (*SUPT5H, CTDP1, EXOSC7, and RBM38*), intracellular trafficking and protein homeostasis (*CHMP1A, FBXL18, and ZDHHC3*), and cellular signaling pathways associated with stress adaptation and environmental responses (*DUSP5, EDNRA, and AKT1*).

Model 2 functional annotation of Top 50 CpG (Table S3), which includes the types of violence specified, was predominantly clustered in neurodevelopmental and synaptic processes (*GPR6, SYNJ2, LRP1, GPM6A, AKT1, SKOR1, MAST1, IQSEC1, TRAPPC5*, and *RPTOR*), intracellular trafficking and cytoskeletal organization (*MYO1E, IQSEC1, UBL3, RANBP10, CCDC40*, and *CHMP1A*), genomic stability and DNA damage responses (*FAAP24, EME2, TELO2, MDM2, CTDP1*, and *CDC6*), and immune‐inflammatory regulation (*P2RX7, HLA‐DRA, TRIM31*, and *ABCF1*).

## 4 Discussion

This study observed gender and racial disparities, with girls, and Black and Brown participants exhibiting higher exposure to family violence. This aligns with literature suggesting that structural and historical inequalities contribute to disproportionate exposure to adversity (Brodsky and Cattaneo 2013; Williams et al. 2019). Moreover, some authors explain that boys’ ability to defend themselves, on the basis of their greater physical strength and socialization, may explain the higher incidence of violence among girls. Furthermore, gender inequalities are historically structured by domination and oppression built into social relations, across social classes, races, ethnicities, and age groups (Pinto Junior et al. 2015; Riba and Zioni 2022). These findings should be interpreted within broader social and cultural contexts, and causal interpretations should be avoided.

Regarding the co‐occurrence of family violence, most studies have examined different forms of victimization independently. Our emphasis lies on the overlap of various victimizations within the same individual (Chan 2017; Edleson et al. 2007). The consequences of developing amid community violence and inequalities arising from structural factors such as race, gender a culture based on the acceptance of violence as a form of communication, and the occurrence of polyvictimization in different contexts have been associated in the literature with disruptions in executive functioning and later‐life risk for psychiatric disorders and social impairments (Diamond 2013; Stover et al. 2012).

This study also identified differentially methylated CpG sites associated with ED in young adults (Hypothesis i), suggesting an association of genes potentially involved in neural plasticity and cognitive regulation, which may be related to dysfunctions in emotional regulation, planning, problem‐solving, and self‐motivation in young individuals exposed to violent environments. However, given the observational design and the assessment of DNA methylation at a single time point, no causal or temporal inferences can be established.

The observed association between methylation signals linked to ED in youth and exposure to family and community violence during childhood (Hypothesis ii) can be interpreted cautiously within an integrated conceptual perspective. The findings align with prior research indicating that early‐life adversities, particularly within the family environment, can alter neurodevelopmental processes through epigenetic mechanisms (Provençal and Binder 2015; Zannas and West 2014). From an ecological viewpoint (Dahlberg and Krug 2006; World Health Organization 2002), violence represents a multilevel environmental exposure embedded in family and community contexts, often co‐occurring with experiences of deprivation such as neglect or reduced cognitive stimulation. Within the DMAP, threat and deprivation are theorized to influence psychopathology through distinct mechanisms—threat via disrupted emotional processing, and deprivation via impaired cognitive development, including executive functioning (McLaughlin et al. 2014; Larkin and Shelleby 2026). In contexts of cumulative adversities, where threat and deprivation exposures may overlap, distinguishing these pathways empirically remains challenging. This overlap underscores the complex ways in which multiple forms of adversity interact with neurodevelopmental processes across the life course. Together, these frameworks provide a theoretical basis to hypothesize that exposure to violence may become biologically embedded through mechanisms such as DNA methylation, potentially relating to alterations in executive functioning during development.

A major biological theme emerging from the identified genes involves neurodevelopmental and synaptic processes. Model 1 shows that genes, such as *GPR6, SYNJ2, LRP1, GPM6A, GDPD5*, and *AKT1*, are implicated in neuronal differentiation, neurite outgrowth, synaptic signaling, receptor trafficking, and neural plasticity. These processes are essential for the maturation and maintenance of large‐scale neural networks supporting executive functions, including attention, inhibitory control, working memory, and cognitive flexibility. Alterations in synaptic plasticity and neuronal connectivity have been consistently associated with the long‐term neurobiological consequences of early‐life adversity and stress exposure. *AKT1* signaling, *LRP1*‐mediated receptor trafficking, and *GPM6A*‐dependent structural plasticity contribute to mechanisms underlying learning, adaptive behavior, and cognitive performance. Disruptions in these pathways have also been linked to psychiatric disorders characterized by ED and impaired cognitive control (Arnsten 2009; Forrest et al. 2018; Tau and Peterson 2010).

A second cluster encompasses genes involved in genome maintenance, transcriptional regulation, and cellular responses to stress, including *HLTF, FAAP24, TELO2, EME2, MDM2, SUPT5H, CTDP1, RBM38*, and *DUSP5*. These genes participate in DNA repair, replication fork stability, chromatin regulation, RNA processing, and signaling pathways that coordinate cellular adaptation to environmental challenges. Growing evidence indicates that chronic stress and exposure to adversity can influence epigenetic regulation and DNA damage‐response pathways, potentially affecting neuronal resilience and long‐term cognitive functioning. Furthermore, MAPK/ERK signaling regulated by *DUSP5* and p53‐associated pathways regulated by *MDM2* play important roles in neuronal survival, synaptic plasticity, and adaptive responses to stress. Together, these mechanisms provide a plausible biological framework linking environmental exposures to persistent alterations in brain function and executive performance through changes in genomic stability, transcriptional regulation, and cellular homeostasis (Madabhushi et al. 2014; McEwen 2017; Menke and Binder 2014).

In Model 2, genes were predominantly clustered in neurodevelopmental and synaptic processes (*GPR6, SYNJ2, LRP1, GPM6A, AKT1, SKOR1, MAST1, IQSEC1, TRAPPC5*, and *RPTOR*), intracellular trafficking and cytoskeletal organization (*MYO1E, IQSEC1, UBL3, RANBP10, CCDC40*, and *CHMP1A*), genomic stability and DNA damage responses (*FAAP24, EME2, TELO2, MDM2, CTDP1*, and *CDC6*), and immune‐inflammatory regulation (*P2RX7, HLA‐DRA, TRIM31*, and *ABCF1*), strengthening pathways related to neuronal plasticity, membrane trafficking, neuroimmune signaling, and cellular stress adaptation. It reinforces the convergence between neurodevelopment, neuronal plasticity, intracellular trafficking, and neuroinflammation, a set of pathways highly plausible for studies involving violence, early adversity, brain function, and executive functions (Forrest et al. 2018; Tau and Peterson 2010).

This article contributes by suggesting methylation alterations tied to trauma‐related variables such as family and community violence. To the best of our understanding, our previous review (Lacerda et al. 2025) indicated a scarcity in the literature of research linking violence, ED, and DNA methylation, standing out some molecular pathways implicated in cognitive alterations, such as *NR3C1, FKBP5, BDNF*, and *OXTR*. In this article we investigated these genes and additionally identified other CpGs that may warrant further investigation in independent samples.

Notably, CpGs overlapping across ED, family violence, and community violence models (Figures S2 and S3) mapped to genes involved in neuronal signaling and synaptic plasticity (*AGAP3*), neurodevelopment and neural progenitor proliferation (*CDK5RAP2*), transcriptional and posttranscriptional regulation (*SUPT5H and RBPMS*), mitochondrial network maintenance (*MSTO1*/*MSTO2P*), DNA repair and genomic stability (*HLTF*), cell signaling and proliferative responses (*HRCT1*), and ciliary organization and function (*CFAP61*). Collectively, these genes converge on biological processes related to cellular adaptation, neuronal development, and neural plasticity. Although exploratory, these findings are consistent with the involvement of stress‐responsive molecular systems and suggest that pathways related to glutamatergic neurotransmission, calcium‐dependent signaling, and cellular resilience may represent potential targets through which early‐life adversity influences epigenetic regulation (Francis et al. 2025). Furthermore, *NR3C1* gene (Figure S10) is known to be related to stress and cognitive processes (Agorastos et al. 2019; Ferrer et al. 2025), which was associated with ED and physical family violence in our study.

Some loci annotated to genes identified in the study may represent candidates for further investigation. For example, to date, *UNKL* has biological functions in RAC (Ras‐related C3 botulinum toxin substrate) signaling (which affects how cells move, shape, and growth, especially in neurons and immune cells) and ubiquitination regulation (which affects how proteins are processed or destroyed). *UNKL* and its paralogue Unk have been suggested to be involved in cognitive flexibility (Vinsland et al. 2021) and brain development and function (GeneCards n.d.). Importantly, the functional interpretation of these CpGs should be considered exploratory, as their direct biological effects and tissue‐specific relevance remain to be established.

The use of saliva‐derived DNA in epigenome‐wide association studies merits careful consideration, given both its practical advantages and its distinct biological properties. Relative to whole blood, saliva‐derived samples often exhibits slightly lower global DNA methylation levels at many loci (Lowe et al. 2013); however, genome‐wide methylation patterns across these tissues show a high degree of concordance (Braun et al. 2019; Smith et al. 2015). Importantly, well‐characterized exposure‐associated loci display consistent behavior across tissues. These considerations underscore the importance of appropriately accounting for cellular heterogeneity in saliva‐based EWAS, while supporting saliva as a valid biospecimen (Bruinsma et al. 2018; Thompson et al. 2013). Nevertheless, methylation patterns identified in saliva should be interpreted as peripheral biomarkers that may not directly reflect brain‐specific epigenetic processes.

It should be noted that epigenetic variation is understood as reflecting biological embedding of experience rather than deterministic mechanisms. Understanding the epigenetic consequences of adversity is essential from both developmental and transgenerational standpoints. The concept of epigenetic plasticity offers promising directions for early prevention. For example, therapeutic strategies, targeting methylation changes linked to PTSD symptoms, have shown potential in reversing maladaptive patterns (Carleial et al. 2021; Pizzimenti and Lattal 2015; Yehuda et al. 2013). These findings reinforce the need to incorporate psychosocial risk factors—particularly exposure to violence into prenatal and developmental care frameworks, recognizing that maternal and early childhood well‐being have profound implications for long‐term mental and cognitive health across generations. However, evidence regarding the reversibility of specific methylation patterns in humans remains limited and was not assessed in the present study.

Some emphasis should be given to the consequences of impaired executive functioning, which may include difficulties in resolving complex situations, inhibiting inappropriate responses, regulating behavior, adapting to new rules or perspectives, and engaging in goal‐directed planning (Cruz et al. 2020; Goldstein et al. 2014; Jackson et al. 2014; Kofler et al. 2019). These challenges may be further exacerbated in contexts of family and community violence (Carvalho et al. 2020) and have been associated with impulsivity, externalizing behaviors, and violations of social norms (Crowell et al. 2003; Hoaken et al. 2007). However, Op Den Kelder et al. (2018) highlight that the relationship between trauma exposure and executive functioning is likely bidirectional. Although traumatic experiences may hinder the development of executive functions, preexisting executive difficulties may also increase vulnerability to subsequent trauma exposure. Children with lower executive functioning are more prone to behavioral problems, which can elevate their risk of interpersonal trauma, including child maltreatment and community violence, particularly when they are perceived as more difficult to manage within family or school environments (Schoemaker et al. 2013). These dynamics underscore the need for integrated public policies that combine violence prevention with early interventions aimed at strengthening executive functioning and promoting healthy developmental environments.

### 4.1 Limitations

The results emphasize the utility of epigenomic profiling in understanding how socio‐environmental stressors become biologically embedded, affecting long‐term mental health trajectories. However, the interpretation of the results must consider important methodological limitations. The relatively small sample size due to attrition over the 16‐year follow‐up, and lack of replication warrant caution in interpreting specific gene‐level associations, reducing statistical power and increasing the risk of false positives. Furthermore, the fitted models may be subject to coefficient instability and potential overfitting. Due to sample size, it was not feasible to include all potential covariates without compromising statistical power: Models were adjusted for sex and epithelial cell composition, but other factors—such as race/ethnicity, age, socioeconomic status and smoking—may also influence the results, highlighting a limitation that future studies with larger cohorts should address. The attrition may also have increased the risk of selection bias, affecting the representativeness of the sample and the generalizability of the findings, and it may have attenuated the variability in the measures, limiting the ability to detect small effects.

Because ED was measured at a single time point, it is not possible to establish temporal ordering, which limits causal inference and raises the possibility of reverse causation. Self‐reported measures of violence and symptoms may have introduced reporting bias, as responses can be influenced by recall, social desirability, or the respondent's emotional state at the time of assessment. In addition, variations in informants across waves and the potential underreporting of certain forms of violence—particularly psychological or family‐based violence—may have contributed to measurement error.

Another limitation of this study is the complexity and volume of the epigenetic results, which involve numerous CpG sites mapped to multiple genes with detailed annotations. Due to space constraints and the early stage of understanding in the field of epigenetics, especially regarding functional implications and their relationship with executive functions. Therefore, only the most relevant findings are highlighted, whereas the complete dataset is provided as Supporting Information providing future studies and in‐depth studies. Moreover, different CpGs and genes resulted from the investigated methods, making it impossible to delve deeper: Although DMP‐based methods assign genes based on proximity to individual CpG sites, DMR analyses capture broader regions that may overlap multiple genes or regulatory elements, leading to differing gene associations. Additionally, emerging candidate genes related to cognition, stress, or violence, such as those identified in the study, should be viewed as exploratory rather than definitive and need to be further validated with functional analyses, gene expression, or replicative studies. The findings, therefore, should be considered exploratory and require replication in independent and larger samples, as well as longitudinal approaches that allow for the evaluation of temporal relationships and causal mechanisms.

Finally, an additional limitation relates to the restricted ability to capture broader biological organization underlying the observed methylation patterns. Although an exploratory pathway analysis was included to support biological interpretation, future studies with larger samples may enable more integrative approaches.

## 5 Conclusion

The integration of epigenetic data with psychosocial measures provides new insight into how family and community violence shape neurodevelopment through lasting molecular changes. Public health policies must address not only the social determinants of violence but also their biological impact, particularly among racially marginalized populations. Promoting early interventions, including trauma‐informed care and programs to support parenting and safe communities, could help mitigate the epigenetic imprint of violence. Further research is essential to validate these biomarkers and inform precision strategies for mental health promotion in high‐risk youth.

## Author Contributions

**Renata Queiroz Ramos**: conceptualization, data curation, methodology, formal analysis, visualization, writing – original draft, writing – review and editing. **Cosme Marcelo Furtado Passos da Silva**: conceptualization, methodology, writing – review and editing, supervision. **Adriane Feijó Evangelista**: data curation, methodology, formal analysis, writing – original draft, writing – review and editing. **Fernanda Serpeloni**: conceptualization, methodology, writing – original draft, writing – review and editing. **Natasha Reis Lacerda**: writing – original draft, writing – review and editing. **Joviana Quintes Avanci**: writing – original draft, writing – review and editing, funding acquisition. **Simone Gonçalves de Assis**: conceptualization, data curation, methodology, visualization, writing – original draft, writing – review and editing, supervision, funding acquisition. All authors have read and approved the final version submitted and take public responsibility for all aspects of the work.

## Funding

Research supported by National Council for Scientific and Technological Development—CNPq, (grant number 407977/2021‐0) and FAPERJ—Fundação Carlos Chagas Filho de Amparo à Pesquisa do Estado do Rio de Janeiro (E‐26/204.258/2024). R.Q.R. acknowledges support from the Coordenação de Aperfeiçoamento de Pessoal de Nível Superior—Brazil (CAPES)—Finance Code 001.

## Ethics Statement

This study was approved by the Ethics Committee of the Oswaldo Cruz Foundation (CAAE 18723119.0.0000.5240).

## Consent

All participants provided written informed consent or assent, as appropriate.

## Conflicts of Interest

The authors declare no conflicts of interest.

## Generative AI Statement

Artificial intelligence tools were used to support the translation and linguistic refinement of the manuscript from Portuguese to English.

## Supporting information




**Table S1**. Socioeconomic characteristics of participants retained in the cohort.
**Figure S1**. Spearman correlation between executive dysfunctions (ED), family violence (FamV), psychological family violence (PsyFV), physical family violence (PhyFV), and community violence (ComV).
**Figure S2. Overlap of the top 10,000 CpGs associated with executive dysfunction (ED), family violence (FamV), and community violence (ComV)**. Nine CpGs overlapped in all variables: (i) cg22315619—*HRCT1* (histidine rich carboxyl terminus 1 putative regulator of ERBB2/MAPK‐related signaling associated with cell proliferation, migration, and tumor progression); (ii) cg22122068—*SUPT5H* (SPT5 homolog, DSIF elongation factor subunit—regulates RNA polymerase II transcription elongation, promoter‐proximal pausing, mRNA processing, capping, and transcriptional responses to cellular stimuli.); (iii) cg04073618—*MSTO1*/*MSTO2P* (*MSTO1*: Misato mitochondrial distribution and morphology regulator 1). *MSTO1* regulates mitochondrial morphology, distribution, fusion dynamics, and maintenance of mitochondrial networks; *MSTO2P* is a pseudogene with uncertain biological function; (iv) cg02619478—*RBPMS* (RNA binding protein with multiple splicing—involved in mRNA processing, alternative splicing, RNA transport, posttranscriptional regulation, and neuronal development); (v) cg26151310—*HLTF* (helicase‐like transcription factor) involved in DNA damage tolerance, chromatin remodeling, replication fork restart, genomic stability, and post‐replication DNA repair; template switching during DNA damage tolerance; (vi) cg16129515 ‐ *CDK5RAP2* (CDK5 regulatory subunit‐associated protein 2) involved in microtubule organization, centrosome maturation, spindle assembly, neurogenesis, and neural progenitor proliferation; (vii) cg26949393—*AGAP3 *(ArfGAP with GTPase domain, ankyrin repeat, and PH domain 3) multifunctional signaling protein involved in NMDA receptor signaling, AMPA receptor trafficking, synaptic plasticity, Ras/ERK signaling, Arf6‐mediated membrane trafficking, and neuronal signal transduction; and (viii) cg04674519 ‐ *CFAP61* (Cilia and flagella associated protein 61, structural component of motile cilia and flagella involved in axonemal organization, sperm flagellar motility, and ciliary function); (ix) cg08745216, not annotated.
**Figure S3. Overlap of the top 10,000 CpGs associated with executive dysfunction (ED), psychological violence (PsyFV), physical violence (PhyFV), and community violence (ComV)**. Only one CpG overlapped between all variables when including physical and psychological violence in the family: (i) cg01619156, not annotated.
**Table S2. Top 50 CpGs in Model 1: Executive dysfunctions (ED), family violence (FamV), and community violence (ComV)**, adjusted by sex and epithelial cell composition.
**Table S3. Top 50 CpGs in Model 2: Executive dysfunctions (ED), psychological family violence (PsyFV), Physical family violence (PhyFV), and community violence (ComV)**, adjusted by sex and epithelial cell composition.
**Figure S4. Linear regression models of differential methylation (beta values) at CpG site cg02023548 (*MRGPRD*) across (a) ED; (b) FamV; (c) PsyFV; (d) PhyFV; and (e) ComV**.
**Figure S5. Linear regression models of differential methylation (beta values) at CpG site cg27352156 (*UNKL*) across (a) ED; (b) FamV; (c) PsyFV; (d) PhyFV; and (e) ComV**.
**Figure S6. Linear regression models of differential methylation (beta values) at CpG site cg20927656 (*DPPA3*) across (a) ED; (b) FamV; (c) PsyFV; (d) PhyFV; and (e) ComV**.
**Figure S7. Linear regression models of differential methylation (beta values) at CpG site cg15667844 (*DUSP5*) across (a) ED; (b) FamV; (c) PsyFV; (d) PhyFV; and (e) ComV**.
**Figure S8. Linear regression models of differential methylation (beta values) at CpG site cg08390865 (*GPR6*) across (a) ED; (b) FamV; (c) PsyFV; (d) PhyFV; and (e) ComV**.
**Figure S9. Linear regression models of differential methylation (beta values) at CpG site cg14553549 (*CAPN13*) across (a) ED; (b) FamV; (c) PsyFV; (d) PhyFV; and (e) ComV**.
**Figure S10. Linear regression models of differential methylation (beta values) at CpG site cg08845721 (*NR3C1*) across (a) ED; (b) FamV; (c) PsyFV; (d) PhyFV; and (e) ComV**.
**Figure S11. Linear regression models of differential methylation (beta values) at CpG site cg10300814 (*FKBP5*) across (a) ED; (b) FamV; (c) PsyFV; (d) PhyFV; and (e) ComV**.

## Data Availability

The data and code supporting this study are available upon reasonable request, with measures in place to preserve personally identifiable information.

## References

[dev70183-bib-0001] Agorastos, A. , P. Pervanidou , G. P. Chrousos , and D. G. Baker . 2019. “Developmental Trajectories of Early Life Stress and Trauma: A Narrative Review on Neurobiological Aspects Beyond Stress System Dysregulation.” Frontiers in Psychiatry 10: 118. 10.3389/fpsyt.2019.00118. 30914979 PMC6421311

[dev70183-bib-0002] Ainamani, H. E. , T. Elbert , D. K. Olema , and T. Hecker . 2017. “PTSD Symptom Severity Relates to Cognitive and Psycho‐Social Dysfunctioning—A Study With Congolese Refugees in Uganda.” European Journal of Psychotraumatology 8: 1283086. 10.1080/20008198.2017.1283086. 28326164 PMC5328389

[dev70183-bib-0003] Alvarez, J. A. , and E. Emory . 2006. “Executive Function and the Frontal Lobes: A Meta‐Analytic Review.” Neuropsychology Review 16: 17–42. 10.1007/s11065-006-9002-x. 16794878

[dev70183-bib-0004] Arnsten, A. F. T. 2009. “Stress Signalling Pathways That Impair Prefrontal Cortex Structure and Function.” Nature Reviews Neuroscience 10: 410–422. 10.1038/nrn2648. 19455173 PMC2907136

[dev70183-bib-0005] Assis, S. G. , R. V. C. Oliveira , J. Q. Avanci , et al. 2011. “Child Mental Health: Social Inequalities and Family Resources.” In Social Determinants and Mental Health. Nova Science Publishers.

[dev70183-bib-0006] Aston‐Jones, G. , and J. D. Cohen . 2005. “An Integrative Theory of Locus Coeruleus‐Norepinephrine Function: Adaptive Gain and Optimal Performance.” Annual Review of Neuroscience 28: 403–450. 10.1146/annurev.neuro.28.061604.135709. 16022602

[dev70183-bib-0007] Avanci, J. , S. Assis , R. Oliveira , and T. Pires . 2012. “CHILDHOOD DEPRESSION. Exploring the Association Between Family Violence and Other Psychosocial Factors in Low‐Income Brazilian Schoolchildren.” Child and Adolescent Psychiatry and Mental Health 6: 26. 10.1186/1753-2000-6-26. 22776354 PMC3413564

[dev70183-bib-0008] Baddeley, A. 2010. “Working Memory.” Current Biology 20: R136–R140. 10.1016/j.cub.2009.12.014. 20178752

[dev70183-bib-0009] Barekatain, M. , L. C. Johansson , J. H. Lam , et al. 2024. “Structural Insights Into the High Basal Activity and Inverse Agonism of the Orphan Receptor GPR6 Implicated in Parkinson's Disease.” Science Signaling 17: eado8741. 10.1126/scisignal.ado8741. 39626010 PMC11850111

[dev70183-bib-0010] Barkley, R. A. 2011. Barkley Deficits in Executive Functioning Scale (BDEFS for Adults). Guilford Press.

[dev70183-bib-0011] Bogliacino, F. , G. Grimalda , P. Ortoleva , and P. Ring . 2017. “Exposure to and Recall of Violence Reduce Short‐Term Memory and Cognitive Control.” Proceedings of the National Academy of Sciences 114: 8505–8510. 10.1073/pnas.1704651114. PMC5559026 28739904

[dev70183-bib-0012] Braun, P. R. , S. Han , B. Hing , et al. 2019. “Genome‐Wide DNA Methylation Comparison Between Live Human Brain and Peripheral Tissues Within Individuals.” Translational Psychiatry 9: 47. 10.1038/s41398-019-0376-y. 30705257 PMC6355837

[dev70183-bib-0013] Brazilian Institute of Geography and Statistics [IBGE] . 2022. Demographic Census . Brazilian Institute of Geography and Statistics.

[dev70183-bib-0014] Brodsky, A. E. , and L. B. Cattaneo . 2013. “A Transconceptual Model of Empowerment and Resilience: Divergence, Convergence and Interactions in Kindred Community Concepts.” American Journal of Community Psychology 52: 333–346. 10.1007/s10464-013-9599-x. 24057948

[dev70183-bib-0015] Bronfenbrenner, U. 1977. “Toward an Experimental Ecology of Human Development.” American Psychologist 32: 513–531. 10.1037/0003-066X.32.7.513.

[dev70183-bib-0016] Bruinsma, F. J. , J. E. Joo , E. M. Wong , G. G. Giles , and M. C. Southey . 2018. “The Utility of DNA Extracted From Saliva for Genome‐Wide Molecular Research Platforms.” BMC Research Notes 11: 8. 10.1186/s13104-017-3110-y. 29310721 PMC5759806

[dev70183-bib-0017] Bücker, J. , F. Kapczinski , R. Post , et al. 2012. “Cognitive Impairment in School‐Aged Children With Early Trauma.” Comprehensive Psychiatry 53: 758–764. 10.1016/j.comppsych.2011.12.006. 22300905

[dev70183-bib-0018] Carleial, S. , D. Nätt , E. Unternährer , et al. 2021. “DNA Methylation Changes Following Narrative Exposure Therapy in a Randomized Controlled Trial With Female Former Child Soldiers.” Scientific Reports 11: 18493. 10.1038/s41598-021-98067-9. 34531495 PMC8445994

[dev70183-bib-0019] Carvalho, J. N. , A. M. Renner , J. C. Donat , T. C. de Moura , R. P. Fonseca , and C. H. Kristensen . 2020. “Executive Functions and Clinical Symptoms in Children Exposed to Maltreatment.” Applied Neuropsychology: Child 9: 1–12. 10.1080/21622965.2018.1497989. 30295547

[dev70183-bib-0020] Champagne, F. A. 2010. “Epigenetic Influence of Social Experiences Across the Lifespan.” Developmental Psychobiology 52: 299–311. 10.1002/dev.20436. 20175106

[dev70183-bib-0021] Champagne, F. A. , and J. P. Curley . 2011. “Epigenetic Influence of the Social Environment.” In Brain, Behavior and Epigenetics, edited by A Petronis and J Mill , 185–208. Springer Berlin Heidelberg.

[dev70183-bib-0022] Chan, K. L. 2017. “Family Polyvictimization and Elevated Levels of Addiction and Psychopathology Among Parents in a Chinese Household Sample.” Journal of Interpersonal Violence 32: 2433–2452. 10.1177/0886260515592617. 26130685

[dev70183-bib-0023] Chan, M. H. , M. Meijer , S. M. Merrill , et al. 2026. “Not all Saliva Samples Are Equal: The Role of Cellular Heterogeneity in DNA Methylation and Epigenetic Age Analyses With Biological and Psychosocial Factors.” Psychoneuroendocrinology 184: 107688. 10.1016/j.psyneuen.2025.107688. 41265020 PMC13328854

[dev70183-bib-0024] Chen, M. A. , A. S. LeRoy , M. Majd , et al. 2021. “Immune and Epigenetic Pathways Linking Childhood Adversity and Health Across the Lifespan.” Frontiers in Psychology 12: 788351. 10.3389/fpsyg.2021.788351. 34899540 PMC8662704

[dev70183-bib-0025] Clinchard, C. , B. Casas , and J. Kim‐Spoon . 2024. “Child Maltreatment and Executive Function Development Throughout Adolescence and Into Young Adulthood.” Development and Psychopathology 37: 1889–1902. 10.1017/S0954579424001457. 39465607 PMC12035961

[dev70183-bib-0026] Cordeiro, A. S. , and C. A. S. M. Minervino . 2024. “Impacto do Isolamento Social Provocado pela Covid‐19 nas Funções Executivas de Crianças.” Avaliação Psicológica 23: 287–294.

[dev70183-bib-0027] Correia, S. S. 2013. Bateria de Avaliação Neuropsicológica de Coimbra (BANC): Estudo de validação em crianças e adolescentes institucionalizados vítimas de maus tratos . UC/FPCE.

[dev70183-bib-0028] Crowell, T. A. , K. M. Kieffer , S. Kugeares , and R. D. Vanderploeg . 2003. “Executive and Nonexecutive Neuropsychological Functioning in Antisocial Personality Disorder.” Cognitive and Behavioral Neurology 16: 100–109. 10.1097/00146965-200306000-00003. 12799596

[dev70183-bib-0029] Cruz, A. R. , A. De Castro‐Rodrigues , and F. Barbosa . 2020. “Executive Dysfunction, Violence and Aggression.” Aggression and Violent Behavior 51: 101380. 10.1016/j.avb.2020.101380.

[dev70183-bib-0030] Cunningham, F. , J. E. Allen , J. Allen , et al. 2022. “Ensembl 2022.” Nucleic Acids Research 50: D988–D995. 10.1093/nar/gkab1049. 34791404 PMC8728283

[dev70183-bib-0031] Dahlberg, L. L. , and E. G. Krug . 2006. “Violence a Global Public Health Problem.” Ciência & Saúde Coletiva 11: 277–292. 10.1590/S1413-81232006000200007.

[dev70183-bib-0032] Diamond, A. 2013. “Executive Functions.” Annual Review of Psychology 64: 135–168. 10.1146/annurev-psych-113011-143750. PMC4084861 23020641

[dev70183-bib-0033] Dias, É. B. 2019. Marcos desenvolvimentais das funções executivas na infância: um estudo com crianças em idade escolar [Doctoral dissertation, Federal University of Paraiba] . Institutional Repository of the Federal University of Paraiba (UFPB). https://repositorio.ufpb.br/jspui/handle/123456789/19426

[dev70183-bib-0034] dos Santos Oliveira, N. C. , S. Katrinli , S. G. de Assis , et al. 2023. “Community and Domestic Violence Are Associated With DNA Methylation GrimAge Acceleration and Heart Rate Variability in Adolescents.” European Journal of Psychotraumatology 14: 2202054. 10.1080/20008066.2023.2202054. 37144662 PMC10165931

[dev70183-bib-0035] Dos Santos Oliveira, N. C. , F. Serpeloni , and S. Gonçalves De Assis . 2023. “The Interplay Between DNA Methylation and Cardiac Autonomic System Functioning: A Systematic Review.” International Journal of Environmental Health Research 33: 54–70. 10.1080/09603123.2021.2000590. 34753378

[dev70183-bib-0036] Du, P. , X. Zhang , C.‐C. Huang , et al. 2010. “Comparison of Beta‐Value and M‐Value Methods for Quantifying Methylation Levels by Microarray Analysis.” BMC Bioinformatics [Electronic Resource] 11: 587. 10.1186/1471-2105-11-587. 21118553 PMC3012676

[dev70183-bib-0037] Edleson, J. L. , A. L. Ellerton , E. A. Seagren , S. L. Kirchberg , S. O. Schmidt , and A. T. Ambrose . 2007. “Assessing Child Exposure to Adult Domestic Violence.” Children and Youth Services Review 29: 961–971. 10.1016/j.childyouth.2006.12.009.

[dev70183-bib-0038] Ferrer, A. , J. Labad , N. Salvat‐Pujol , et al. 2025. “Genetic and Epigenetic Changes to the Glucocorticoid Receptor Gene (NR3C1) and Cognition in Major Depressive Disorder.” Spanish Journal of Psychiatry and Mental Health Advance online publication 25: 000183. 10.1016/j.sjpmh.2024.12.002. 40189105

[dev70183-bib-0039] Finkelhor, D. , R. K. Ormrod , and H. A. Turner . 2007. “Poly‐Victimization: A Neglected Component in Child Victimization.” Child Abuse & Neglect 31: 7–26. 10.1016/j.chiabu.2006.06.008. 17224181

[dev70183-bib-0040] Forrest, M. P. , E. Parnell , and P. Penzes . 2018. “Dendritic Structural Plasticity and Neuropsychiatric Disease.” Nature Reviews Neuroscience 19: 215–234. 10.1038/nrn.2018.16. 29545546 PMC6442683

[dev70183-bib-0041] Francis, A. , L. A. McKibben , and Y. Dwivedi . 2025. “Early‐Life Adversity–Induced Epigenetic Reprogramming of Prefrontal Cortex in Rats Subjected to Maternal Separation.” Biological Psychiatry Global Open Science 5: 100487. 10.1016/j.bpsgos.2025.100487. 40342557 PMC12060456

[dev70183-bib-0042] Franklin, T. B. , B. J. Saab , and I. M. Mansuy . 2012. “Neural Mechanisms of Stress Resilience and Vulnerability.” Neuron 75: 747–761. 10.1016/j.neuron.2012.08.016. 22958817

[dev70183-bib-0043] GeneCards . n. d. *UNK Gene—Unk Zinc Finger*. GeneCards: The human gene database. Weizmann Institute of Science. https://www.genecards.org/card/UNKL

[dev70183-bib-0044] Glaser, D. 2002. “Emotional Abuse and Neglect (Psychological Maltreatment): A Conceptual Framework.” Child Abuse & Neglect 26: 697–714. 10.1016/S0145-2134(02)00342-3. 12201163

[dev70183-bib-0045] Godoy, V. P. , F. G. D. Mata , B. R. Conde , et al. 2015. “Brazilian Portuguese Transcultural Adaptation of Barkley Deficits in Executive Functioning Scale (BDEFS).” Archives of Clinical Psychiatry (São Paulo) 42: 147–152. 10.1590/0101-60830000000065.

[dev70183-bib-0046] Godoy, V. P. , P. Mattos , and L. F. Malloy‐Diniz . 2018. BDEFS—Escala de Avaliação de Disfunções Executivas de Barkley. Hogrefe.

[dev70183-bib-0047] Goldstein, S. , J. A. Naglieri , and D. Princiotta . 2014. “Introduction: A History of Executive Functioning as a Theoretical and Clinical Construct.” In Handbook of Executive Functioning, edited by S Goldstein and JA Naglieri , 3–12. Springer New York.

[dev70183-bib-0048] Harrison, P. W. , M. R. Amode , O. Austine‐Orimoloye , et al. 2024. “Ensembl 2024.” Nucleic Acids Research 52: D891–D899. 10.1093/nar/gkad1049. 37953337 PMC10767893

[dev70183-bib-0049] Hasselmann, M. H. , and M. E. Reichenheim . 2003. “Adaptação Transcultural da Versão em português da Conflict Tactics Scales Form R (CTS‐1), Usada para Aferir Violência no Casal: Equivalências Semântica e de Mensuração.” Cad Saúde Pública 19: 1083–1093. 10.1590/S0102-311x2003000400030. 12973573

[dev70183-bib-0050] Haushofer, J. , and E. Fehr . 2014. “On the Psychology of Poverty.” Science 344: 862–867. 10.1126/science.1232491. 24855262

[dev70183-bib-0051] Hoaken, P. N. S. , D. B. Allaby , and J. Earle . 2007. “Executive Cognitive Functioning and the Recognition of Facial Expressions of Emotion in Incarcerated Violent Offenders, Non‐Violent Offenders, and Controls.” Aggressive Behavior 33: 412–421. 10.1002/ab.20194. 17683105

[dev70183-bib-0052] Illumina . 2020. Infinium MethylationEPIC v1.0 B5 Manifest File (CSV Format) . Illumina.

[dev70183-bib-0053] Irizarry, R. A. , and M. I. Love . 2025. *Package ‘rafalib’*: Convenience Functions for Routine Data Exploration. https://cran.r-project.org/web/packages/rafalib/index.html.

[dev70183-bib-0054] Jackson, C. J. , N. J. Loxton , P. Harnett , J. Ciarrochi , and M. J. Gullo . 2014. “Original and Revised Reinforcement Sensitivity Theory in the Prediction of Executive Functioning: A Test of Relationships Between Dual Systems.” Personality and Individual Differences 56: 83–88. 10.1016/j.paid.2013.08.024.

[dev70183-bib-0055] Kofler, M. J. , L. N. Irwin , E. F. Soto , N. B. Groves , S. L. Harmon , and D. E. Sarver . 2019. “Executive Functioning Heterogeneity in Pediatric ADHD.” Journal of Abnormal Child Psychology 47: 273–286. 10.1007/s10802-018-0438-2. 29705926 PMC6204311

[dev70183-bib-0056] Lacerda, N. R. , R. Q. Ramos , F. Serpeloni , et al. 2025. Epigenetics, Early‐Life Adversities, and Cognitive Function: A Scoping Review. Fundação Oswaldo Cruz.

[dev70183-bib-0057] Lake, D. , S. A. L. Corrêa , and J. Müller . 2016. “Negative Feedback Regulation of the ERK1/2 MAPK Pathway.” Cellular and Molecular Life Sciences 73: 4397–4413. 10.1007/s00018-016-2297-8. 27342992 PMC5075022

[dev70183-bib-0058] Larkin, K. , and E. C. Shelleby . 2026. “Testing the Dimensional Model of Adversity and Psychopathology: The Mediating Roles of Executive Functioning and Emotion Regulation.” Research on Child and Adolescent Psychopathology 54: 31. 10.1007/s10802-026-01433-2. 41688621

[dev70183-bib-0059] Letkiewicz, A. M. , C. J. Funkhouser , and S. A. Shankman . 2021. “Childhood Maltreatment Predicts Poorer Executive Functioning in Adulthood Beyond Symptoms of Internalizing Psychopathology.” Child Abuse & Neglect 118: 105140. 10.1016/j.chiabu.2021.105140. 34098377 PMC8292220

[dev70183-bib-0060] Lowe, R. , C. Gemma , H. Beyan , et al. 2013. “Buccals Are Likely to Be a More Informative Surrogate Tissue Than Blood for Epigenome‐Wide Association Studies.” Epigenetics 8: 445–454. 10.4161/epi.24362. 23538714 PMC3674053

[dev70183-bib-0061] Luna, B. , S. Marek , B. Larsen , B. Tervo‐Clemmens , and R. Chahal . 2015. “An Integrative Model of the Maturation of Cognitive Control.” Annual Review of Neuroscience 38: 151–170. 10.1146/annurev-neuro-071714-034054. PMC5661874 26154978

[dev70183-bib-0062] Madabhushi, R. , L. Pan , and L.‐H. Tsai . 2014. “DNA Damage and Its Links to Neurodegeneration.” Neuron 83: 266–282. 10.1016/j.neuron.2014.06.034. 25033177 PMC5564444

[dev70183-bib-0063] Malik, N. M. 2008. “Exposure to Domestic and Community Violence in a Nonrisk Sample.” Journal of Interpersonal Violence 23: 490–504. 10.1177/0886260507312945. 18272724

[dev70183-bib-0064] Mani, A. , S. Mullainathan , E. Shafir , and J. Zhao . 2013. “Poverty Impedes Cognitive Function.” Science 341: 976–980. 10.1126/science.1238041. 23990553

[dev70183-bib-0065] Margari, L. , F. Craig , F. Margari , A. Legrottaglie , R. Palumbi , and C. De Giambattista . 2016. “A Review of Executive Function Deficits in Autism Spectrum Disorder and Attention‐Deficit/Hyperactivity Disorder.” Neuropsychiatric Disease and Treatment 12: 1191–1202. 10.2147/NDT.S104620. 27274255 PMC4869784

[dev70183-bib-0066] Marques, N. M. 2015. *Fatores clínicos e de risco associados ao desempenho cognitivo em crianças vítimas de abuso sexual* [Master's thesis, Universidade de São Paulo]. Biblioteca Digital de Teses e Dissertações da Universidade de São Paulo. 10.11606/D.47.2015.tde-25092015-105920.

[dev70183-bib-0067] Martins, E. , and H. Szymanski . 2004. “A Abordagem Ecológica de Urie Bronfenbrenner em Estudos com Famílias.” Estudos e Pesquisas em Psicologia 4: 63–77.

[dev70183-bib-0068] McEwen, B. S. 2017. “Neurobiological and Systemic Effects of Chronic Stress.” Chronic Stress 1: 2470547017692328. 10.1177/2470547017692328. 28856337 PMC5573220

[dev70183-bib-0069] McLaughlin, K. A. , M. A. Sheridan , and H. K. Lambert . 2014. “Childhood Adversity and Neural Development: Deprivation and Threat as Distinct Dimensions of Early Experience.” Neuroscience & Biobehavioral Reviews 47: 578–591. 10.1016/j.neubiorev.2014.10.012. 25454359 PMC4308474

[dev70183-bib-0070] Meixiong, J. , and X. Dong . 2017. “Mas‐Related G Protein–Coupled Receptors and the Biology of Itch Sensation.” Annual Review of Genetics 51: 103–121. 10.1146/annurev-genet-120116-024723. 29178819

[dev70183-bib-0071] Menke, A. , and E. B. Binder . 2014. “Epigenetic Alterations in Depression and Antidepressant Treatment.” Dialogues in Clinical Neuroscience 16: 395–404. 10.31887/DCNS.2014.16.3/amenke. 25364288 PMC4214180

[dev70183-bib-0072] Metwally, E. , H. A. Al‐Abbadi , T. Hussain , G. Murtaza , A. M. Abdellatif , and M. F. Ahmed . 2023. “Calpain Signaling: From Biology to Therapeutic Opportunities in Neurodegenerative Disorders.” Frontiers in Veterinary Science 10: 1235163. 10.3389/fvets.2023.1235163. 37732142 PMC10507866

[dev70183-bib-0073] Middleton, L. Y. M. , J. Dou , J. Fisher , et al. 2022. “Saliva Cell Type DNA Methylation Reference Panel for Epidemiological Studies in Children.” Epigenetics 17: 161–177. 10.1080/15592294.2021.1890874. 33588693 PMC8865319

[dev70183-bib-0074] Morris, T. J. , L. M. Butcher , A. Feber , et al. 2014. “ChAMP: 450k Chip Analysis Methylation Pipeline.” Bioinformatics 30: 428–430. 10.1093/bioinformatics/btt684. 24336642 PMC3904520

[dev70183-bib-0075] National Center for Biotechnology Information [NCBI] . n. d. Gene. National Center for Biotechnology Information. https://www.ncbi.nlm.nih.gov/gene/.

[dev70183-bib-0076] Op Den Kelder, R. , A. L. Van Den Akker , H. M. Geurts , et al. 2018. “Executive Functions in Trauma‐Exposed Youth: A Meta‐Analysis.” European Journal of Psychotraumatology 9: 1450595. 10.1080/20008198.2018.1450595. 33488998 PMC7803075

[dev70183-bib-0077] Passos, A. P. D. 2019. Integração de variáveis motoras, cognitivas, nutricionais, metabólicas e de influência epigenética relativas à Primeira Infância como uma ferramenta para investigação do Desenvolvimento Infantil . Intechopen.

[dev70183-bib-0078] Pinto, L. W. , and S. G. D. Assis . 2013. “Violência Familiar e Comunitária em Escolares do Município de São Gonçalo, Rio de Janeiro, Brasil.” Revista Brasileira de Epidemiologia 16: 288–300. 10.1590/S1415-790x2013000200006. 24142002

[dev70183-bib-0079] Pinto Junior, A. A. , V. Cassepp‐Borges , and J. G. D. Santos . 2015. “Caracterização da Violência Doméstica contra Crianças e Adolescentes e as Estratégias Interventivas em um Município do Estado do Rio de Janeiro, Brasil.” Cad saúde colet 23: 124–131. 10.1590/1414-462x201500020062.

[dev70183-bib-0080] Pires, T. O. , C. da Silva , and S. G. de Assis . 2013. “Association Between Family Environment and Attention Deficit Hyperactivity Disorder in Children—Mothers' and Teachers' Views.” BMC Psychiatry 13: 215. 10.1186/1471-244X-13-215. 23978164 PMC3765901

[dev70183-bib-0081] Pizzimenti, C. L. , and K. M. Lattal . 2015. “Epigenetics and Memory: Causes, Consequences and Treatments for Post‐Traumatic Stress Disorder and Addiction.” Genes, Brain and Behavior 14: 73–84. 10.1111/gbb.12187. 25560936 PMC4526190

[dev70183-bib-0082] Provençal, N. , and E. B. Binder . 2015. “The Effects of Early Life Stress on the Epigenome: From the Womb to Adulthood and Even Before.” Experimental Neurology 268: 10–20. 10.1016/j.expneurol.2014.09.001. 25218020

[dev70183-bib-0083] Radtke, K. M. , M. Ruf , H. M. Gunter , et al. 2011. “Transgenerational Impact of Intimate Partner Violence on Methylation in the Promoter of the Glucocorticoid Receptor.” Translational Psychiatry 1: e21–e21. 10.1038/tp.2011.21. 22832523 PMC3309516

[dev70183-bib-0084] Riba, A. C. , and F. Zioni . 2022. “O Corpo da Criança como Receptáculo da Violência Física: Análise dos dados epidemiológicos do Viva/Sinan.” Saúde em Debate 46: 193–207. 10.1590/0103-11042022e516.

[dev70183-bib-0085] Richters, J. E. , and P. Martinez . 1993. “The Nimh Community Violence Project: I. Children as Victims of and Witnesses to Violence.” Psychiatry 56: 7–21. 10.1080/00332747.1993.11024617. 8488215

[dev70183-bib-0086] Ritchie, M. E. , B. Phipson , D. Wu , et al. 2015. “Limma Powers Differential Expression Analyses for RNA‐Sequencing and Microarray Studies.” Nucleic Acids Research 43: e47–e47. 10.1093/nar/gkv007. 25605792 PMC4402510

[dev70183-bib-0087] Roberts, R. J. , and B. F. Pennington . 1996. “An Interactive Framework for Examining Prefrontal Cognitive Processes.” Developmental Neuropsychology 12: 105–126. 10.1080/87565649609540642.

[dev70183-bib-0088] Romero‐Martínez, Á. , M. Lila , S. Vitoria‐Estruch , and L. Moya‐Albiol . 2021. “Can Attention and Working Memory Impairments of Intimate Partner Perpetrators Explain Their Risky Decision Making?” Journal of Interpersonal Violence 36: NP6492–NP6507. 10.1177/0886260518814263. 30499368

[dev70183-bib-0089] Schiele, M. A. , and K. Domschke . 2018. “Epigenetics at the Crossroads Between Genes, Environment and Resilience in Anxiety Disorders.” Genes, Brain and Behavior 17: e12423. 10.1111/gbb.12423. 28873274

[dev70183-bib-0090] Schoemaker, K. , H. Mulder , M. Deković , and W. Matthys . 2013. “Executive Functions in Preschool Children With Externalizing Behavior Problems: A Meta‐Analysis.” Journal of Abnormal Child Psychology 41: 457–471. 10.1007/s10802-012-9684-x. 23054130

[dev70183-bib-0091] Serpeloni, F. , D. Nätt , S. G. D. Assis , E. Wieling , and T. Elbert . 2020. “Experiencing Community and Domestic Violence Is Associated With Epigenetic Changes in DNA Methylation of BDNF and CLPX in Adolescents.” Psychophysiology 57: e13382. 10.1111/psyp.13382. 31059136 PMC7003421

[dev70183-bib-0092] Serpeloni, F. , K. Radtke , S. G. de Assis , F. Henning , D. Nätt , and T. Elbert . 2017. “Grandmaternal Stress During Pregnancy and DNA Methylation of the Third Generation: An Epigenome‐Wide Association Study.” Translational Psychiatry 7: e1202–e1202. 10.1038/tp.2017.153. 28809857 PMC5611722

[dev70183-bib-0093] Shields, A. E. 2017. “Epigenetic Signals of How Social Disadvantage “Gets Under the Skin”: A Challenge to the Public Health Community.” Epigenomics 9: 223–229. 10.2217/epi-2017-0013. 28234017

[dev70183-bib-0094] Silva Filho, O. C. D. , J. Q. Avanci , T. D. O. Pires , R. de Vasconcellos Carvalhaes Oliveira , and S. G. Assis . 2023. “Attachment, Suicidal Behavior, and Self‐Harm in Childhood and Adolescence: A Study of a Cohort of Brazilian Schoolchildren.” BMC Pediatrics 23: 403. 10.1186/s12887-023-04215-7. 37592202 PMC10433545

[dev70183-bib-0095] Simons, D. A. , and S. K. Wurtele . 2010. “Relationships Between Parents' Use of Corporal Punishment and Their Children's Endorsement of Spanking and Hitting Other Children.” Child Abuse & Neglect 34: 639–646. 10.1016/j.chiabu.2010.01.012. 20638720

[dev70183-bib-0096] Smith, A. K. , V. Kilaru , T. Klengel , et al. 2015. “DNA Extracted From Saliva for Methylation Studies of Psychiatric Traits: Evidence Tissue Specificity and Relatedness to Brain.” American Journal of Medical Genetics Part B: Neuropsychiatric Genetics 168: 36–44. 10.1002/ajmg.b.32278. PMC4610814 25355443

[dev70183-bib-0122] Stelzer, G. , N. Rosen , I. Plaschkes , et al. 2016. “The GeneCards Suite: From Gene Data Mining to Disease Genome Sequence Analyses.” Current Protocols in Bioinformatics 54, no. 1: 1.30.1–1.30.33. 10.1002/cpbi.5. 27322403

[dev70183-bib-0097] Stover, C. S. , A. Urdahl , and C. Easton . 2012. “Depression as a Mediator of the Association Between Substance Abuse and Negative Parenting of Fathers.” American Journal of Drug and Alcohol Abuse 38: 344–349. 10.3109/00952990.2011.649221. 22243417 PMC3640321

[dev70183-bib-0098] Straus, M. A. 1979. “Measuring Intrafamily Conflict and Violence: The Conflict Tactics (CT) Scales.” Journal of Marriage and the Family 41: 75–88. 10.2307/351733.

[dev70183-bib-0099] Straus, M. A. , and R. J. Gelles . 1995. Physical Violence in American Families: Risk Factors and Adaptations to Violence in 8,145 Families. Transaction Publishers.

[dev70183-bib-0100] Straus, M. A. , S. L. Hamby , D. Finkelhor , D. W. Moore , and D. Runyan . 1998. “Identification of Child Maltreatment With the Parent‐Child Conflict Tactics Scales: Development and Psychometric Data for a National Sample of American Parents.” Child Abuse & Neglect 22: 249–270. 10.1016/S0145-2134(97)00174-9. 9589178

[dev70183-bib-0101] Stuss, D. T. , and M. P. Alexander . 2000. “Executive Functions and the Frontal Lobes: A Conceptual View.” Psychological Research 63: 289–298. 10.1007/s004269900007. 11004882

[dev70183-bib-0102] Tau, G. Z. , and B. S. Peterson . 2010. “Normal Development of Brain Circuits.” Neuropsychopharmacology 35: 147–168. 10.1038/npp.2009.115. 19794405 PMC3055433

[dev70183-bib-0103] Teicher, M. H. , and J. A. Samson . 2016. “Annual Research Review: Enduring Neurobiological Effects of Childhood Abuse and Neglect.” Journal of Child Psychology and Psychiatry 57: 241–266. 10.1111/jcpp.12507. 26831814 PMC4760853

[dev70183-bib-0104] Thompson, T. M. , D. Sharfi , M. Lee , C. M. Yrigollen , O. Y. Naumova , and E. L. Grigorenko . 2013. “Comparison of Whole‐Genome DNA Methylation Patterns in Whole Blood, Saliva, and Lymphoblastoid Cell Lines.” Behavior Genetics 43: 168–176. 10.1007/s10519-012-9579-1. 23269419 PMC3577999

[dev70183-bib-0105] Tian, Y. , T. J. Morris , A. P. Webster , et al. 2017. “ChAMP: Updated Methylation Analysis Pipeline for Illumina BeadChips.” Bioinformatics 33: 3982–3984. 10.1093/bioinformatics/btx513. 28961746 PMC5860089

[dev70183-bib-0106] Toriyama, K. , W. K. Au Yeung , A. Inoue , et al. 2024. “DPPA3 Facilitates Genome‐Wide DNA Demethylation in Mouse Primordial Germ Cells.” BMC Genomics [Electronic Resource] 25: 344. 10.1186/s12864-024-10192-7. 38580899 PMC10996186

[dev70183-bib-0107] Turner, H. A. , D. Finkelhor , and R. Ormrod . 2010. “Poly‐Victimization in a National Sample of Children and Youth.” American Journal of Preventive Medicine 38: 323–330. 10.1016/j.amepre.2009.11.012. 20171535

[dev70183-bib-0108] Vinsland, E. , P. Baskaran , S. R. Mihaylov , et al. 2021. “The Zinc Finger/RING Domain Protein Unkempt Regulates Cognitive Flexibility.” Scientific Reports 11: 16299. 10.1038/s41598-021-95286-y. 34381067 PMC8357790

[dev70183-bib-0109] Weaver, I. C. G. , N. Cervoni , F. A. Champagne , et al. 2004. “Epigenetic Programming by Maternal Behavior.” Nature Neuroscience 7: 847–854. 10.1038/nn1276. 15220929

[dev70183-bib-0110] Weinbach, N. , and A. Henik . 2012. “The Relationship Between Alertness and Executive Control.” Journal of Experimental Psychology: Human Perception and Performance 38: 1530–1540. 10.1037/a0027875. 22468726

[dev70183-bib-0111] Wen, S. , J. Zhu , X. Han , et al. 2024. “Childhood Maltreatment and Risk of Endocrine Diseases: An Exploration of Mediating Pathways Using Sequential Mediation Analysis.” BMC Medicine 22: 59. 10.1186/s12916-024-03271-9. 38331807 PMC10854183

[dev70183-bib-0112] Williams, D. R. , J. A. Lawrence , B. A. Davis , and C. Vu . 2019. “Understanding How Discrimination Can Affect Health.” Health Services Research 54: 1374–1388. 10.1111/1475-6773.13222. 31663121 PMC6864381

[dev70183-bib-0113] Wolfe, D. A. 2018. “Why Polyvictimization Matters.” Journal of Interpersonal Violence 33: 832–837. 10.1177/0886260517752215. 29411694

[dev70183-bib-0114] World Health Organization . 2002. World Report on Violence and Health. World Health Organization—WHO.

[dev70183-bib-0115] World Health Organization . 2016. INSPIRE: Seven Strategies for Ending Violence Against Children. World Health Organization.

[dev70183-bib-0116] Ximenes, L. F. , S. G. D. Assis , T. D. O. Pires , and J. Q. Avanci . 2013. “Violência Comunitária e Transtorno de Estresse Pós‐traumático em Crianças e Adolescentes.” Psicologia: Reflexão e Crítica 26: 443–450. 10.1590/S0102-79722013000300003.

[dev70183-bib-0117] Yaros, A. , J. E. Lochman , and K. Wells . 2016. “Parental Aggression as a Predictor of Boys' Hostile Attribution Across the Transition to Middle School.” International Journal of Behavioral Development 40: 452–458. 10.1177/0165025415607085. 27647945 PMC5026323

[dev70183-bib-0118] Yehuda, R. , N. P. Daskalakis , F. Desarnaud , et al. 2013. “Epigenetic Biomarkers as Predictors and Correlates of Symptom Improvement Following Psychotherapy in Combat Veterans With PTSD.” Frontiers in Psychiatry 4: 118. 10.3389/fpsyt.2013.00118. 24098286 PMC3784793

[dev70183-bib-0119] Yuen, E. Y. , J. Wei , and Z. Yan . 2017. “Molecular and Epigenetic Mechanisms for the Complex Effects of Stress on Synaptic Physiology and Cognitive Functions.” International Journal of Neuropsychopharmacology 20: 948–955. 10.1093/ijnp/pyx052. 29016816 PMC5737802

[dev70183-bib-0120] Zannas, A. S. , and A. E. West . 2014. “Epigenetics and the Regulation of Stress Vulnerability and Resilience.” Neuroscience 264: 157–170. 10.1016/j.neuroscience.2013.12.003. 24333971 PMC3959582

[dev70183-bib-0121] Zelazo, P. D. , and S. M. Carlson . 2012. “Hot and Cool Executive Function in Childhood and Adolescence: Development and Plasticity.” Child Development Perspectives 6: 354–360. 10.1111/j.1750-8606.2012.00246.x.

