# Supplementary material for: Executive Function and Epigenetic Markers in Youth Exposed to Family and Community Violence in Childhood
Source: Dev Psychobiol. 2026 Aug 2;68(5):e70183. doi: 10.1002/dev.70183 (PMC13430299; doi:10.1002/dev.70183)
Supplement: Supplementary file 1 — Table S1. Socioeconomic characteristics of participants retained in the cohort. Figure S1. Spearman correlation between executive dysfunctions (ED), family violence (FamV), psychological family violence (PsyFV), physical family violence (PhyFV), and community violence (ComV). Figure S2. Overlap of the top 10,000 CpGs associated with executive dysfunction (ED), family violence (FamV), and community violence (ComV). Nine CpGs overlapped in all variables: (i) cg22315619—HRCT1 (histidine rich carboxyl terminus 1 putative regulator of ERBB2/MAPK‐related signaling associated with cell proliferation, migration, and tumor progression); (ii) cg22122068—SUPT5H (SPT5 homolog, DSIF elongation factor subunit—regulates RNA polymerase II transcription elongation, promoter‐proximal pausing, mRNA processing, capping, and transcriptional responses to cellular stimuli.); (iii) cg04073618—MSTO1/MSTO2P (MSTO1: Misato mitochondrial distribution and morphology regulator 1). MSTO1 regulates mitochondrial morphology, distribution, fusion dynamics, and maintenance of mitochondrial networks; MSTO2P is a pseudogene with uncertain biological function; (iv) cg02619478—RBPMS (RNA binding protein with multiple splicing—involved in mRNA processing, alternative splicing, RNA transport, posttranscriptional regulation, and neuronal development); (v) cg26151310—HLTF (helicase‐like transcription factor) involved in DNA damage tolerance, chromatin remodeling, replication fork restart, genomic stability, and post‐replication DNA repair; template switching during DNA damage tolerance; (vi) cg16129515 ‐ CDK5RAP2 (CDK5 regulatory subunit‐associated protein 2) involved in microtubule organization, centrosome maturation, spindle assembly, neurogenesis, and neural progenitor proliferation; (vii) cg26949393—AGAP3 (ArfGAP with GTPase domain, ankyrin repeat, and PH domain 3) multifunctional signaling protein involved in NMDA receptor signaling, AMPA receptor trafficking, synaptic plasticity, Ras/ERK signaling, Arf [file DEV-68-e70183-s001.docx]

**Executive Function and Epigenetic Markers in Youth Exposed to Family and Community Violence in Childhood**

Renata Queiroz Ramos^1,2*^; Cosme Marcelo Furtado Passos da Silva^3^; Adriane Feijó Evangelista^4^, Fernanda Serpeloni^1^; Natasha Reis Lacerda^1^; Joviana Quintes Avanci^1^; Simone Gonçalves de Assis^1*^

**Supplementary material**

**Table S1**. Socioeconomic characteristics of participants retained in the cohort.

| **Variables** | **2005**  **(Baseline)** | **2022**  **(Final sample analyzed)** |
| --- | --- | --- |
| N (Retained) | 500 | 78 |
| % Sex (Male) | 51.6% | 44.9% |
| % Age under 9 years in the first grade of elementary school at the beginning of the study | 74.6% | 89.7% |
| % Race/Skin Color (Non-white) | 66.3% | 73.7% |
| % Social stratum (Lower) | 96.5% | 82.5% |
|  |  |  |

**
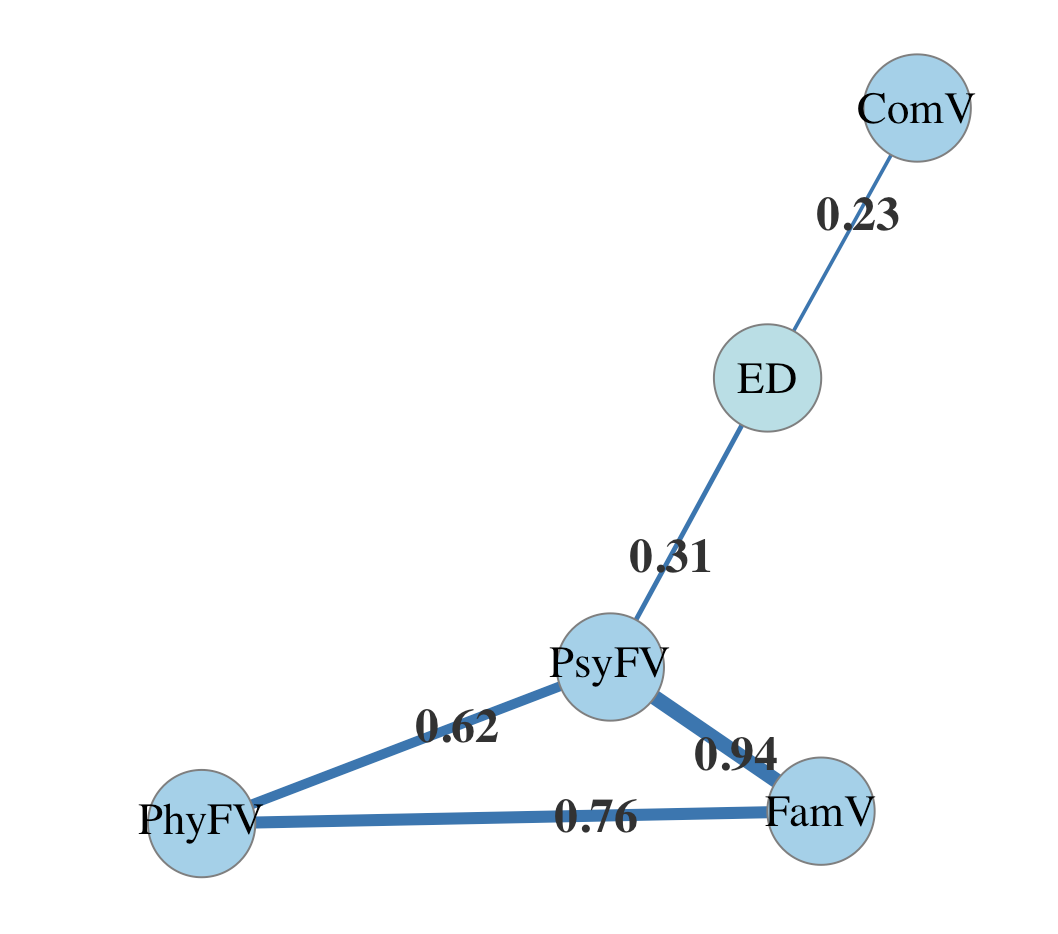
**

**Figure S1**. Spearman correlation between Executive Dysfunctions (ED), Family Violence (FamV), Family Psychological Violence (PsyFV), Family Physical Violence (PhyFV) and Community Violence (ComV).


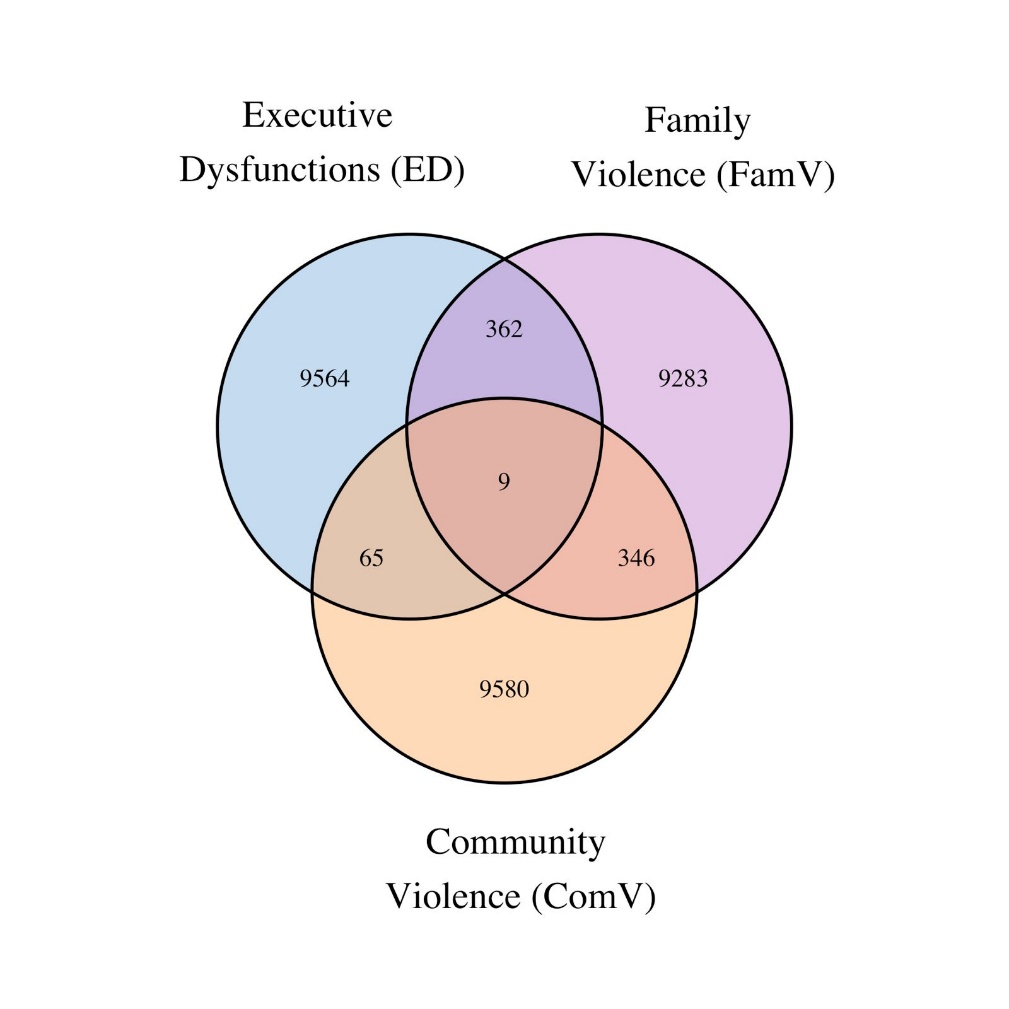


**Figure S2**. **Overlap of the Top 10,000 CpGs Associated with Executive Dysfunction (ED), Family Violence (FamV) and Community Violence (ComV).** 9 CpGs overlapped in all variables: (i) cg22315619 - *HRCT1* (Histidine rich carboxyl terminus 1 Putative regulator of ERBB2/MAPK-related signaling associated with cell proliferation, migration, and tumor progression; (ii) cg22122068 - *SUPT5H* (SPT5 homolog, DSIF elongation factor subunit - regulates RNA polymerase II transcription elongation, promoter-proximal pausing, mRNA processing, capping, and transcriptional responses to cellular stimuli.); (iii) cg04073618 - *MSTO1/MSTO2P* (MSTO1: Misato mitochondrial distribution and morphology regulator 1). MSTO1 regulates mitochondrial morphology, distribution, fusion dynamics, and maintenance of mitochondrial networks; MSTO2P is a pseudogene with uncertain biological function; (iv) cg02619478 - *RBPMS* (RNA binding protein with multiple splicing - involved in mRNA processing, alternative splicing, RNA transport, post-transcriptional regulation and neuronal development); (v) cg26151310 – *HLTF* (Helicase-like transcription factor) involved in DNA damage tolerance, chromatin remodeling, replication fork restart, genomic stability, and post-replication DNA repair; template switching during DNA damage tolerance; (vi) cg16129515 - *CDK5RAP2* (CDK5 regulatory subunit-associated protein 2) involved in microtubule organization, centrosome maturation, spindle assembly, neurogenesis, and neural progenitor proliferation; (vii) cg26949393 - *AGAP3* (ArfGAP with GTPase domain, ankyrin repeat and PH domain 3) Multifunctional signaling protein involved in NMDA receptor signaling, AMPA receptor trafficking, synaptic plasticity, Ras/ERK signaling, Arf6-mediated membrane trafficking, and neuronal signal transduction; and (viii) cg04674519 - *CFAP61* (Cilia and flagella associated protein 61 - Structural component of motile cilia and flagella involved in axonemal organization, sperm flagellar motility, and ciliary function.); (ix) cg08745216, not annotated.

**
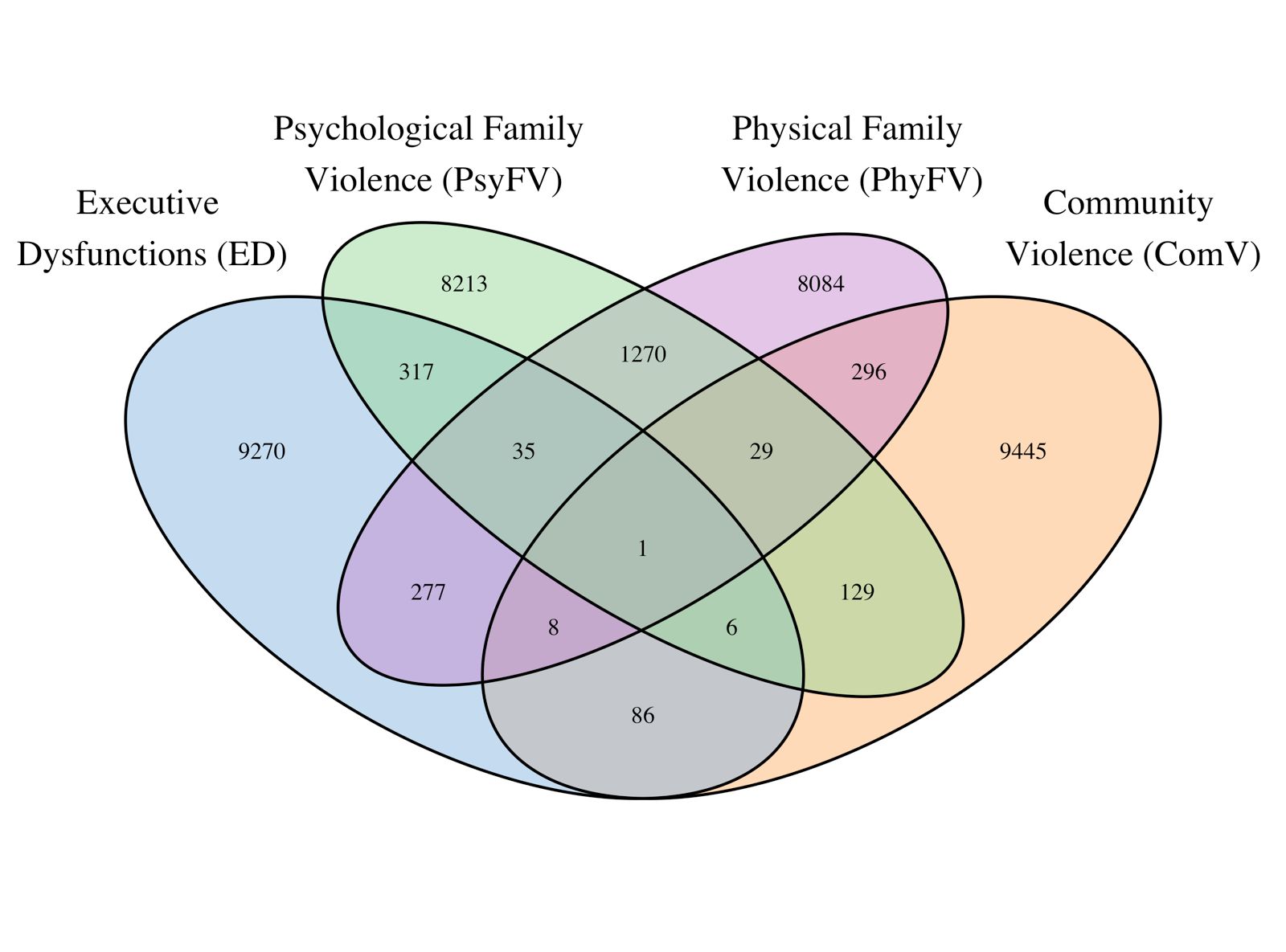
**

**Figure S3**. **Overlap of the Top 10,000 CpGs Associated with Executive Dysfunction (ED), Psychological Violence (PsyFV), Physical Violence (PhyFV), and Community Violence (ComV)**. Only one CpG overlapped between all variables when including physical and psychological violence in the family: (i) cg01619156, not annotated.

**Table S2**. **Top 50 CpGs in Model 1: Executive Dysfunctions (ED), Family Violence (FamV) and Community Violence (ComV)**, adjusted by sex and epithelial cell composition.

| **CpG probe** | **logFC** | **Chr** | **Position** | **Relation to Gene Group** | **Relation to CpG Island** | **P-value** | **Adj.P.Val^1^** | **Gene** | **Gene name** | **Gene functions and pathways^2^** |
| --- | --- | --- | --- | --- | --- | --- | --- | --- | --- | --- |
| ***Executive Dysfunction + Family Violence + Community Violence, Sex and Cell type proportions*** | | | | | | | | | | |
| cg02023548 | 0,40209845 | 11 | 68748461 | TSS200 | - | p<0.001 | **0.001** | *MRGPRD* | Mas Related GPR Family Member D | G protein-coupled receptor predominantly expressed in sensory neurons; involved in nociceptive signaling, itch perception, and responses to peripheral sensory stimuli. Involved in the signaling pathway of the receptor related to angiotensin-mediated vasodilation, which plays a role in the regulation of systemic blood pressure. |
| cg20927656 | -1,218593966 | 12 | 7863229 | TSS1500 | - | p<0.001 | **0.045** | *DPPA3* | Developmental pluripotency-associated protein 3 | Protects epigenetic marks during early embryonic reprogramming; involved in maintenance of DNA methylation at imprinted loci, pluripotency, and germ-cell development. required for intracellular trafficking and cleavage. |
| cg27352156 | 0,404270217 | 16 | 1416292 | Body | S_Shore | p<0.001 | **0.045** | *UNKL* | Unkempt like zinc finger protein | Encodes a protein that may be involved in the regulation of cell differentiation. Possible regulation of cellular stress. |
| cg08390865 | -0,520240206 | 6 | 110299446 | TSS200 | Island | p<0.001 | **0.049** | *GPR6* | G protein-coupled receptor 6 | Implicated in dopaminergic neurotransmission and motor behavior. Related to learning. Involvement in pathways related to neuronal signaling, synaptic transmission, and neurodevelopment. |
| cg15667844 | 0,59705582 | 10 | 112256729 | TSS1500 | N_Shore | p<0.001 | **0.049** | *DUSP5* | Dual specificity phosphatase 5 | Negative regulator of the MAPK/ERK signaling cascade involved in proliferation, differentiation, and cellular responses to environmental stimuli. |
| cg02872136 | -0,701457788 | 10 | 43725302 | TSS200; 5'UTR | Island | p<0.001 | 0.064 | *RASGEF1A* | RasGEF domain family member 1A | Involvement in pathways related to neuronal signaling, synaptic transmission, and neurodevelopment. |
| cg18004235 | 0,360563434 | 2 | 19808330 | - | - | p<0.001 | 0.086 | *-* | - | Has been studied for its role in cancer. |
| cg23779604 | 1,084848158 | 7 | 2760784 | - | S_Shelf | 0.11 | 0.086 | *AMZ1* | Archaelysin family metallopeptidase 1 | Predicted to enable metal ion binding activity and metallopeptidase activity. Predicted to be involved in proteolysis. |
| cg27609554 | 0,75919273 | 6 | 158417144 | Body | - | 0.11 | 0.086 | *SYNJ2* | synaptojanin 2 | Diseases associated include Neurodevelopmental Disorder with Cerebellar Atrophy and Motor Dysfunction. Involvement in pathways related to neuronal signaling, synaptic transmission, and neurodevelopment. |
| cg00974629 | 0,419316922 | 4 | 148401903 | TSS200 | N_Shore | p<0.001 | 0.091 | *EDNRA* | Endothelin receptor type A | Endothelin-1 receptor that regulates vasoconstriction, vascular homeostasis, craniofacial and cardiovascular development, cellular proliferation, and stress-responsive signaling pathways. |
| cg01004278 | -0,610680433 | 5 | 132148978 | TSS200 | Island | p<0.001 | 0.091 | *ANKRD43* | Ankyrin repeat domain-containing protein 43 | Expression studies suggest roles in neuronal development and cellular differentiation. |
| cg02446475 | 0,643383316 | 6 | 31275807 | - | N_Shore | p<0.001 | 0.091 | *-* | - | - |
| cg03114804 | 0,411474 | 12 | 57599358 | Body | S_Shelf | p<0.001 | 0.091 | *LRP1* | Low-density lipoprotein receptor related protein 1 | It is a multifunctional receptor involved in the endocytosis of various molecules, including lipoproteins and extracellular proteins. It has been associated with migraine. Involvement in pathways related to neuronal signaling, synaptic transmission, and neurodevelopment. |
| cg03190944 | 0,400698618 | 16 | 88388231 | - | - | p<0.001 | 0.091 | - | - | - |
| cg04444394 | -0,476014444 | 12 | 69202020 | TSS1500; 5'UTR; 1stExon | Island | p<0.001 | 0.091 | *MDM2* | MDM2 proto-oncogene | Controls DNA damage responses, apoptosis, cell-cycle progression, senescence, and AKT-p53 signaling. Studied in cancers, glioblastoma, and neural stem cells. |
| cg05318486 | -0,994148431 | 7 | 5553423 | TSS200 | Island | p<0.001 | 0.091 | *FBXL18* | F-box and leucine-rich repeat protein 18 | Involved in ubiquitin-dependent protein degradation and regulation of protein stability. Modulates apoptosis, cell-cycle-related processes, AKT signaling, and protein homeostasis. |
| cg12349416 | 0,340517162 | 11 | 4206098 | - | N_Shelf | p<0.001 | 0.091 | - | - | - |
| cg13964906 | -0,751422931 | 3 | 45016900 | TSS1500;  5'UTR | N_Shore | p<0.001 | 0.091 | *EXOSC7;*  *ZDHHC3* | Exosome component 7/  Zinc finger DHHC-type palmitoyltransferase 3 | Genes involved in post-transcriptional and post-translational regulatory processes, contributing to RNA quality control, protein modification, intracellular trafficking, and synaptic signaling. |
| cg16792604 | -0,672977693 | 6 | 30539044 | TSS200 | Island | p<0.001 | 0.091 | *ABCF1* | ATP-binding cassette subfamily F member 1 | Involved in inflammatory and immune signaling pathways and participates in genome surveillance and transcriptional regulation. |
| cg18210893 | 1,269377398 | 19 | 33468395 | 3'UTR | - | p<0.001 | 0.091 | *FAAP24* | Fanconi anemia core complex-associated protein 24- | DNA repair protein associated with the Fanconi anemia pathway; participates in recognition and repair of DNA interstrand crosslinks and maintenance of genomic stability. |
| cg18591013 | 0,306633126 | 18 | 77464335 | Body | Island | p<0.001 | 0.091 | *CTDP1* | CTD phosphatase subunit 1 | Involved in transcription elongation, RNA processing, genome stability, DNA damage repair, and maintenance of cellular homeostasis. |
| cg19637330 | 1,505857708 | 1 | 19110922 | - | Island | p<0.001 | 0.091 | - | - | - |
| cg20450979 | -0,602006231 | 4 | 176923542 | 5'UTR;  1stExon | Island | p<0.001 | 0.091 | *GPM6A* | Glycoprotein M6A | Neuronal membrane glycoprotein involved in neurite outgrowth, dendritic spine formation, synaptogenesis, neural plasticity, and stress responsiveness. |
| cg03190944 | 0,3260023410 | - | - | - | - | p<0.001 | 0,091 | - | - | - |
| cg13786083 | 1,144084609 | 1 | 19110734 | - | Island | p<0.001 | 0.106 | - | - | - |
| cg19619414 | 0,424953517 | 7 | 218950 | Body | Island | p<0.001 | 0.106 | *FAM20C* | FAM20C golgi associated secretory pathway kinase | Golgi-localized kinase responsible for phosphorylation of secreted proteins; essential for bone and tooth mineralization, extracellular matrix biology, and phosphate homeostasis. |
| cg02752105 | 0,412039627 | 11 | 373615 | Body | N_Shelf | p<0.001 | 0.116 | *B4GALNT4* | Beta-1,4-N-acetyl-galactosaminyltransferase 4 | Iinvolved in N- and O-linked glycan biosynthesis, protein maturation, membrane organization, and cellular signaling. |
| cg00329656 | -0,668062267 | 1 | 17054246 | - | S_Shelf | p<0.001 | 0.136 | - | - | - |
| cg00623111 | 0,420510286 | 16 | 1559692 | Body | N_Shore | p<0.001 | 0.136 | *TELO2* | TEL2 telomere maintenance 2 homolog | Participates in DNA damage responses, genome maintenance, cell-cycle checkpoint control, cellular growth, and neurodevelopment. |
| cg03846249 | -0,44863276 | 12 | 130621750 | - | N_Shore | p<0.001 | 0.136 | - | - | - |
| cg04105597 | 0,660709825 | 1 | 205585639 | 3'UTR | - | p<0.001 | 0.136 | *ELK4* | ETS transcription factor, ELK4 | Involved in immediate-early gene activation, cellular differentiation, proliferation, neuronal plasticity, and adaptive transcriptional responses to environmental stimuli. |
| cg14459293 | 0,475950526 | 13 | 50126394 | Body | - | p<0.001 | 0.136 | *RCBTB1* | RCC1 and BTB domain-containing protein 1 | Participates in chromatin organization, protein ubiquitination pathways, cellular homeostasis, and oxidative stress regulation. |
| cg16393207 | 0,453640833 | 11 | 75237739 | TSS1500 | Island | p<0.001 | 0.136 | *GDPD5* | Glycerophosphodiester phosphodiesterase domain-containing protein 5 | Involved in neurite formation, motor neuron differentiation, Notch pathway regulation, and membrane-associated signaling. Related to neural plasticity and maturation of neural circuits. |
| cg18405900 | -0,325613003 | 6 | 106959549 | TSS200 | N_Shore | p<0.001 | 0.136 | *AIM1*  *CRYBG1* | Absent in melanoma 1 Crystallin beta-gamma domain containing 1 | Cytoskeleton-associated protein containing beta/gamma-crystallin domains; implicated in cell differentiation, adhesion, migration, and tumor suppression. |
| cg26700702 | 0,395529078 | 16 | 1960348 | - | N_Shore | p<0.001 | 0.136 | - | - | - |
| cg27146824 | 0,25146406 | 16 | 1826040 | Body | N_Shore | p<0.001 | 0.136 | *EME2* | Essential meiotic structure-specific endonuclease subunit 2 | Involved in replication fork processing, recombination intermediate resolution, DNA damage signaling, and genome stability maintenance. It contributes to the preservation of genomic integrity in the face of cellular stress. |
| cg00440468 | 1,591440798 | 1 | 19110768 | - | Island | p<0.001 | 0.159 | - | - | - |
| cg03597607 | 0,459975612 | 16 | 89714999 | Body | S_Shore | p<0.001 | 0.159 | *CHMP1A* | Charged multivesicular body protein 1A | Involved in endosomal trafficking, multivesicular body formation, cytokinesis, and lysosomal/autophagic degradation pathways. |
| cg07215697 | 0,431680226 | 6 | 158902287 | Body | - | p<0.001 | 0.159 | *TULP4* | TUB like protein 4 | Possibly involved in intracellular signaling and maintenance of neuronal homeostasis. Studied in glioblastoma, and neural stem cells. |
| cg08925046 | 0,361213375 | 10 | 97008920 | Body | - | p<0.001 | 0.159 | *PDLIM1* | PDZ and LIM domain 1 | Involved in actin organization, cell adhesion, migration, neuronal signaling, and protein-complex assembly. |
| cg17799760 | 0,452016289 | 20 | 55968730 | Body | S_Shore | p<0.001 | 0.159 | *RBM38* | RNA binding motif protein 38 | p53-regulated RNA-binding protein involved in mRNA stability, translational control, cell-cycle regulation, differentiation, and stress responses. |
| cg04526365 | -0,717105784 |  |  |  |  | p<0.001 | 0,1589 | *-* | *-* | *-* |
| cg13614753 | -0,595334426 |  |  |  |  | p<0.001 | 0,1589 | *-* | *-* | *-* |
| cg10027217 | -0,504061091 | 9 | 140082883 | TSS200;  1stExon | Island | p<0.001 | 0.169 | *SSNA1;*  *ANAPC2* | SS nuclear autoantigen1  Anaphase-promoting complex subunit 2 | Participate in fundamental processes of cellular organization and neural development. |
| cg04437881 | 0,585461054 | 17 | 2697740 | - | - | p<0.001 | 0.170 | *RAP1;*  *GAP2* | RAP1 GTPase Activating Protein 2 | Regulator of Rap1 signaling involved in GTPase-mediated signal transduction, vesicular secretion, and intracellular signaling. |
| cg04824553 | -0,665007597 | - | - | - | - | p<0.001 | 0,1701 | *-* | - | - |
| cg01534543 | 0,344626691 | 17 | 59564836 | - | - | p<0.001 | 0.172 | *-* | - | - |
| cg10100767 | 0,381003986 | 14 | 105246561 | Body | - | p<0.001 | 0.172 | *AKT1* | AKT serine/threonine kinase 1 | Regulates a wide variety of cellular functions including cell proliferation, survival, metabolism, and angiogenesis in both normal and malignant cells. Associated with cancers and nervous system malformation. Involvement in pathways related to neuronal signaling, synaptic transmission, and neurodevelopment. |
| cg02121892 | 0,597949917 | 11 | 71174976 | Body | - | p<0.001 | 0.180 | *NADSYN1* | NAD Synthetase 1 | Protein Coding gene. Diseases associated include Vertebral, Cardiac, Renal, And Limb Defects Syndrome 3 and Congenital Vertebral-Cardiac-Renal Anomalies Syndrome. Among its related pathways are superpathway of tryptophan utilization and Metabolism of water-soluble vitamins and cofactors. |
| cg23171972 | 0,551215578 | 8 | 143203477 | - | S_Shore | p<0.001 | 0.180 | *-* | - | - |

**^1^** Adjusted with Benjamini-Hochberg method

**^2^** Ewas Atlas and Reactome pathway annotations were based on exploratory pathway analysis and are provided for biological interpretation purposes.

**Table S3**. **Top 50 CpGs in Model 2: Executive Dysfunctions (ED), Psychological Family Violence (PsyFV), Physical Family Violence (PhyFV) and Community Violence (ComV),** adjusted by sex and epithelial cell composition.

| **CpG probe** | **logFC** | **Chr** | **Position** | **Relation to Gene Group** | **Relation to CpG Island** | **P-value** | **Adj.P.Value^1^** | **Gene** | **Gene name** | **Gene functions and pathways^2^** |
| --- | --- | --- | --- | --- | --- | --- | --- | --- | --- | --- |
| cg02023548 | 0,391498877 | 11 | 68748461 | TSS200 | - | <0.001 | **0.002** | *MRGPRD* | MAS related GPR family member D | G protein-coupled receptor predominantly expressed in sensory neurons; involved in nociceptive signaling, itch perception, and responses to peripheral sensory stimuli.  Involved in the signaling pathway of the receptor related to angiotensin-mediated vasodilation, which plays a role in the regulation of systemic blood pressure. |
| cg14553549 | 0,446997887 | 2 | 30967753 | Body | - | <0.001 | **0.036** | *CAPN13* | Calpain 13 | Involved in regulated proteolysis, cellular stress adaptation, protein remodeling, and intracellular signaling. Studied in relation to cancers, hypertension in pregnancy, and inflammatory diseases. |
| cg15667844 | 0,631843707 | 10 | 112256729 | N_Shore | TSS1500 | <0.001 | 0.073 | *DUSP5* | Dual specificity phosphatase 5 | Negative regulator of the MAPK/ERK signaling cascade involved in proliferation, differentiation, and cellular responses to environmental stimuli. |
| cg27352156 | 0,428053755 | 16 | 1416292 | Body | S_Shore | <0.001 | 0.073 | *UNKL* | Unkempt like zinc finger protein | Encodes a protein that may be involved in the regulation of cell differentiation. Possible regulation of cellular stress. |
| cg02446475 | 0,675723672 | 6 | 31275807 | - | N_Shore | <0.001 | 0.074 | *-* | - | - |
| cg02872136 | -0,681886391 | 10 | 43725302 | TSS200;  5'UTR | Island | <0.001 | 0.074 | *RASGEF1A* | RasGEF domain family member 1A | Involvement in pathways related to neuronal signaling, synaptic transmission, and neurodevelopment. |
| cg05318486 | -1,021865594 | 7 | 5553423 | TSS200 | Island | <0.001 | 0.074 | *FBXL18* | F-box and leucine-rich repeat protein 18 | Involved in ubiquitin-dependent protein degradation and regulation of protein stability. Modulates apoptosis, cell-cycle-related processes, AKT signaling, and protein homeostasis |
| cg18004235 | 0,408700405 | 2 | 19808330 | - | - | <0.001 | 0.074 | *-* | - | Has been studied for its role in cancer. |
| cg18210893 | 1,301244767 | 19 | 33468395 | 3'UTR | - | <0.001 | 0.074 | *FAAP24* | Fanconi anemia core complex-associated protein 24- | DNA repair protein associated with the Fanconi anemia pathway; participates in recognition and repairing DNA interstrand crosslinks and maintenance of genomic stability. |
| cg19619414 | 0,459722393 | 7 | 218950 | Body | Island | <0.001 | 0.074 | *FAM20C* | FAM20C golgi associated secretory pathway kinase | Golgi-localized kinase responsible for phosphorylation of secreted proteins; essential for bone and tooth mineralization, extracellular matrix biology, and phosphate homeostasis. |
| cg20450979 | -0,617634449 | 4 | 176923542 | 5'UTR;  1stExon | Island | <0.001 | 0.074 | *GPM6A* | Glycoprotein M6A | Neuronal membrane glycoprotein involved in neurite outgrowth, dendritic spine formation, synaptogenesis, neural plasticity, and stress responsiveness. |
| cg26544134 | 0,507098599 | 18 | 5892753 | TSS1500 | S_Shore | <0.001 | 0.074 | - | - | - |
| cg27609554 | 0,724288513 | 6 | 158417144 | Body | - | <0.001 | 0.074 | SYNJ2 | synaptojanin 2 | Diseases associated include Neurodevelopmental Disorder with Cerebellar Atrophy and Motor Dysfunction. Involvement in pathways related to neuronal signaling, synaptic transmission, and neurodevelopment. |
| cg13887966 | 0,598242824 | 15 | 59568712 | Body | - | <0.001 | 0.086 | *MYO1E* | Myosin IE | Involved in membrane trafficking, endocytosis, cytoskeletal organization, and cell motility. It is expressed in the brain and studied in inflammatory, allergic, and immunological diseases. |
| cg00974629 | 0,417970415 | 4 | 148401903 | TSS200 | N_Shore | <0.001 | 0.113 | *EDNRA* | Endothelin receptor type A | Endothelin-1 receptor that regulates vasoconstriction, vascular homeostasis, craniofacial and cardiovascular development, cellular proliferation, and stress-responsive signaling pathways. |
| cg02752105 | 0,428220855 | 11 | 373615 | Body | N_Shelf | <0.001 | 0.113 | *B4GALNT4* | Beta-1,4-N-acetyl-galactosaminyltransferase 4 | Involved in N- and O-linked glycan biosynthesis, protein maturation, membrane organization, and cellular signaling. |
| cg03114804 | 0,416957396 | 12 | 57599358 | Body | S_Shelf | <0.001 | 0.113 | *LRP1* | Low-density lipoprotein receptor related protein 1 | Involved in the endocytosis of various molecules, including lipoproteins and extracellular proteins. It has been associated with migraine. Involvement in pathways related to neuronal signaling, synaptic transmission, and neurodevelopment. |
| cg08390865 | -0,504346474 | 6 | 110299446 | TSS200 | Island | <0.001 | 0.121 | *GPR6* | G protein-coupled receptor 6 | Implicated in dopaminergic neurotransmission and motor behavior. Related to learning. Involvement in pathways related to neuronal signaling, synaptic transmission, and neurodevelopment^3^. |
| cg03846249 | -0,4562280785 | 12 | 130621750 | - | N_Shore | <0.001 | 0.132 | *-* | - | - |
| cg23171972 | 0,59357978609 | 8 | 143203477 | - | S_Shore | <0.001 | 0.142 | *-* | - | - |
| cg01004278 | -0,597670485 | 5 | 132148978 | TSS200 | Island | <0.001 | 0.160 | *ANKRD43* | Ankyrin repeat domain-containing protein 43 | Expression studies suggest roles in neuronal development and cellular differentiation. |
| cg07215697 | 0,457006375 | 6 | 158902287 | Body | - | <0.001 | 0.160 | *TULP4* | TUB like protein 4 | Possibly involved in intracellular signaling and maintenance of neuronal homeostasis. Studied in glioblastoma, and neural stem cells. |
| cg18916403 | -0,712907326 | 15 | 68115877 | Body | Island | <0.001 | 0.160 | *SKOR1* | SKI Family Transcriptional Corepressor 1 | Protein Coding gene. Located in dendrite and neuronal cell body. Involved in BMP signaling regulation, neuronal differentiation, and neurodevelopment. |
| cg04444394 | -0,470169368 | 12 | 69202020 | TSS1500;  5'UTR;  1stExon | Island | <0.001 | 0.167 | *MDM2* | MDM2 proto-oncogene | Controls DNA damage responses, apoptosis, cell-cycle progression, senescence, and AKT-p53 signaling. Studied cancer, glioblastoma, and neural stem cells. |
| cg16792604 | -0,636309958 | 6 | 30539044 | TSS200 | Island | <0.001 | 0.167 | *ABCF1* | ATP-binding cassette subfamily F member 1 | Involved in inflammatory and immune signaling pathways and participates in genome surveillance and transcriptional regulation. |
| cg18591013 | 0,31432204 | 18 | 77464335 | Body | Island | <0.001 | 0.167 | *CTDP1* | CTD phosphatase subunit 1 | Involved in transcription elongation, RNA processing, genome stability, DNA damage repair, and maintenance of cellular homeostasis. |
| cg15230985 | 0,442858891 | 17 | 78753887 | Body | - | <0.001 | 0.174 | *RPTOR* | Regulatory Associated Protein of MTOR Complex 1 | Involved in nutrient sensing, protein synthesis, autophagy regulation, cellular growth, metabolism, and neurodevelopment. |
| cg08918709 | 0,415970388 | 6 | 30074219 | Body | S_Shelf | <0.001 | 0.180 | *TRIM31* | Tripartite Motif Containing 31 | Involved in immune regulation, inflammation, protein degradation, autophagy, and cellular stress responses. |
| cg10100767 | 0,40532381 | 14 | 105246561 | Body | - | <0.001 | 0.182 | *AKT1* | AKT serine/threonine kinase 1 | Regulates a wide variety of cellular functions including cell proliferation, survival, metabolism, and angiogenesis in both normal and malignant cells. Associated with cancer and nervous system malformation. Involvement in pathways related to neuronal signaling, synaptic transmission, and neurodevelopment. |
| cg12316290 | 0,430914541 | 13 | 30425969 | TSS1500 | S_Shore | <0.001 | 0.182 | *UBL3* | UBL3 ubiquitin like 3 | Involved in post-translational regulation and protein sorting into small extracellular vesicles/exosomes, contributing to intercellular communication, vesicular trafficking, and cellular homeostasis. |
| cg27146824 | 0,260084816 | 16 | 1826040 | Body | N_Shore | <0.001 | 0.198 | *EME2* | Essential meiotic structure-specific endonuclease subunit 2 | Involved in replication fork processing, recombination intermediate resolution, DNA damage signaling, and genome stability maintenance. It contributes to the preservation of genomic integrity in the face of cellular stress. |
| cg01534543 | 0,351077568 | 17 | 59564836 | - | - | <0.001 | 0.204 | *-* | - | - |
| cg01114989 | 0,386826288 | 16 | 67757411 | 3'UTR | S_Shelf | <0.001 | 0.211 | *RANBP10* | RAN binding protein 10 | Involved in cytoskeletal organization, intracellular trafficking, cell division, and neuronal development. |
| cg00623111 | 0,418797291 | 16 | 1559692 | Body | N_Shore | <0.001 | 0.212 | *TELO2* | TEL2 telomere maintenance 2 homolog | Participates in DNA damage responses, genome maintenance, cell-cycle checkpoint control, cellular growth, and neurodevelopment. |
| cg04105597 | 0,664751005 | 1 | 205585639 | 3'UTR | - | <0.001 | 0.230 | *ELK4* | ETS transcription factor, ELK4 | Involved in immediate-early gene activation, cellular differentiation, proliferation, neuronal plasticity, and adaptive transcriptional responses to environmental stimuli. |
| cg04495313 | 0,385894013 | 4 | 42348136 | - | - | <0.001 | 0.230 | *RP11-63A11.1* | - | Long non-coding RNA (lncRNA), potentially involved in transcriptional and epigenetic regulation. Related to environmental stress. |
| cg24145039 | 0,371312633 | 19 | 12962757 | Body | S_Shelf | <0.001 | 0.230 | *MAST1* | Microtubule Associated Serine/ Threonine Kinase 1 | Involved in neuronal development, cytoskeletal regulation, and intracellular signaling. |
| cg12569216 | 0,382934419 | 17 | 78041214 | Body | S_Shore | <0.001 | 0.239 | *CCDC40* | Coiled-Coil Domain Containing 40 | Protein required for motile cilia assembly, ciliary beating, axonemal organization, and microtubule-dependent cellular functions. |
| cg21408848 | 0,393721467 | 3 | 12949849 | Body | Island | <0.001 | 0.239 | *IQSEC1* | IQ Motif and Sec7 Domain ArfGEF 1 | Involved in membrane trafficking, cytoskeletal remodeling, neuronal development, and cell migration. |
| cg11274962 | -0,645736015 | 19 | 7746796 | 5'UTR | Island | <0.001 | 0.241 | *TRAPPC5* | Trafficking Protein Particle Complex Subunit 5 | Involved in vesicle trafficking, ER-to-Golgi transport, intracellular membrane organization, and neurodevelopment. |
| cg12349416 | 0,332346905 | 11 | 4206098 | - | N_Shelf | <0.001 | 0.254 | *-* | - | - |
| cg26656658 | 0,619462486 | 12 | 121615078 | Body | - | <0.001 | 0.257 | *P2RX7* | Purinergic Receptor P2X 7 | Involved in inflammasome activation, neuroinflammation, innate immunity, and cellular stress signaling. |
| cg15838122 | 0,302167087 | 13 | 111409956 | - | - | <0.001 | 0.265 | *-* | - | - |
| cg02569219 | 0,487205716 | 17 | 2266849 | Body | S_Shore | <0.001 | 0.272 | *SGSM2* | small G protein signaling modulator 2 | Protein Coding gene. Diseases associated with SGSM2 include Schindler Disease and Hermansky-Pudlak Syndrome. |
| cg09442654 | -0,581956968 | 8 | 53477881 | 5'UTR; 1stExon | Island | <0.001 | 0.272 | *FAM150A* | ALK And LTK Ligand 1 | Protein Coding gene. Involved in the signaling pathway of the cell surface receptor tyrosine kinase; positive regulation of the MAPK cascade; and positive regulation of neuronal projection development. It is active in the extracellular space. Studied in cancers, glioblastoma, and neural stem cells. |
| cg20296701 | 0,4332539007 | 6 | 36203818 | - | - | <0.001 | 0,272 | *HLA-DRA* | Major Histocompatibility Complex, Class II, DR Alph | Involved in adaptive immunity, inflammatory signaling, and immune regulation. |
| cg20725670 | 0,839500555 | 17 | 38444322 | 1stExon;  5'UTR | S_Shore | <0.001 | 0,277 | *CDC6* | Cell Division Cycle 6 | Protein Coding gene. Functions as a regulator at the early steps of DNA replication. Diseases associated include Meier-Gorlin Syndrome 5 and Meier-Gorlin Syndrome 1. |
| cg05048002 | -0,50979492 | 16 | 30077837 | 5'UTR | Island | <0.001 | 0,295 | *ALDOA* | Aldolase, Fructose-Bisphosphate A | Involved in energy metabolism, neuronal function, cytoskeletal organization, and cellular stress responses. |
| cg18405900 | -0,331687121 | 6 | 106959549 | TSS200 | N_Shore | <0.001 | 0,295 | *AIM1*  *CRYBG1* | Absent in melanoma 1 Crystallin beta-gamma domain containing 1 | Cytoskeleton-associated protein containing beta/gamma-crystallin domains; implicated in cell differentiation, adhesion, migration, and tumor suppression. |
| cg02443148 | 0,736307377 | 7 | 37533599 | - | - | <0.001 | 0,305 | - | - | - |

1 Adjusted with Benjamini-Hochberg method

2 Ewas Atlas and Reactome pathway annotations were based on exploratory pathway analysis and are provided for biological interpretation purposes.

3 Patrick Oeckl, Bastian Hengerer, Boris Ferger. G-protein coupled receptor 6 deficiency alters striatal dopamine and cAMP concentrations and reduces dyskinesia in a mouse model of Parkinson's disease, Experimental Neurology, Volume 257, 2014, Pages 1-9, ISSN 0014-4886, https://doi.org/10.1016/j.expneurol.2014.04.010.


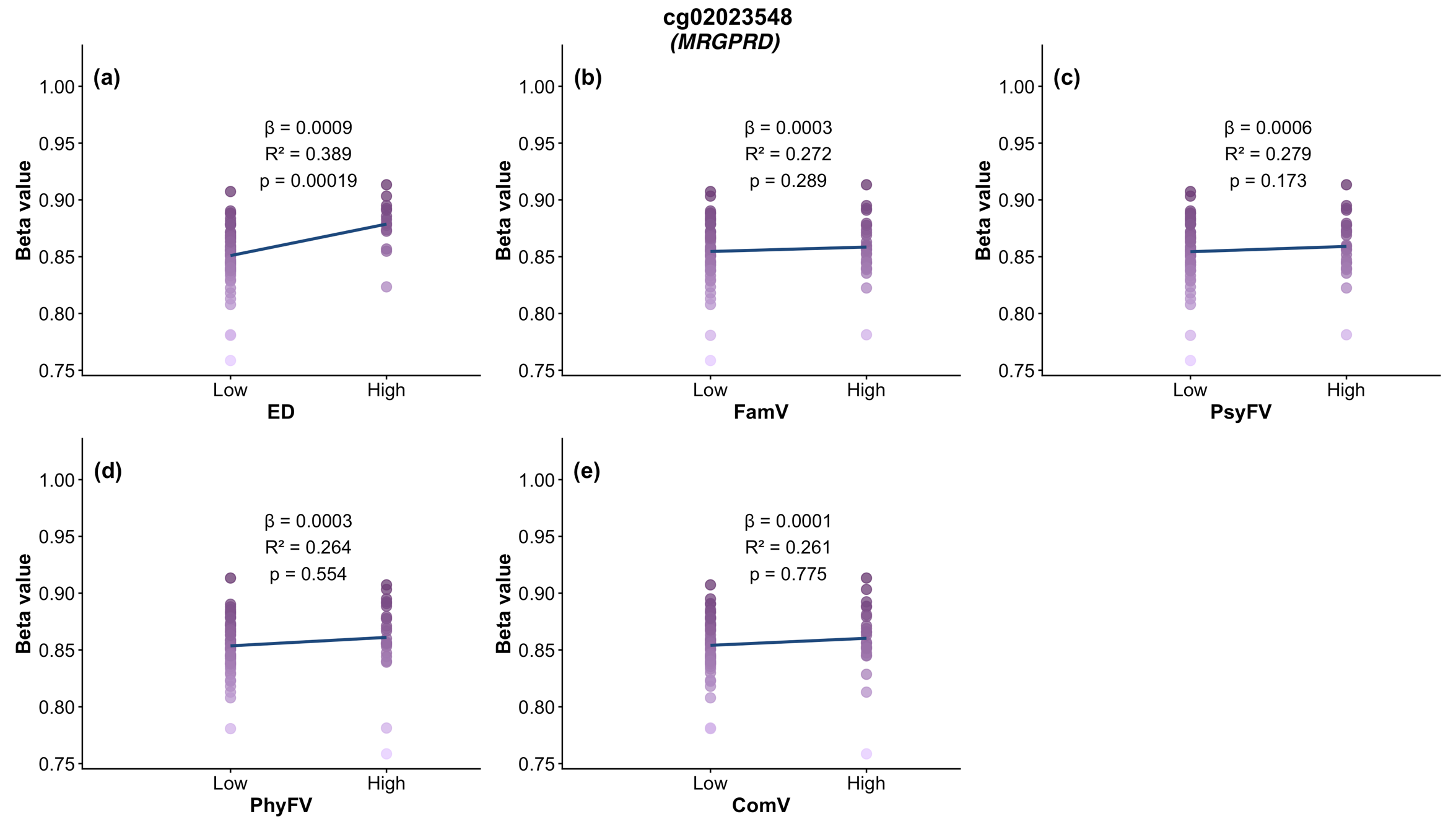


**Figure S4. Linear regression models of differential methylation (beta-values) at CpG site cg02023548 (*MRGPRD*) across (a) ED; (b) FamV; (c) PsyFV; (d) PhyFV; and (e) ComV.**

**
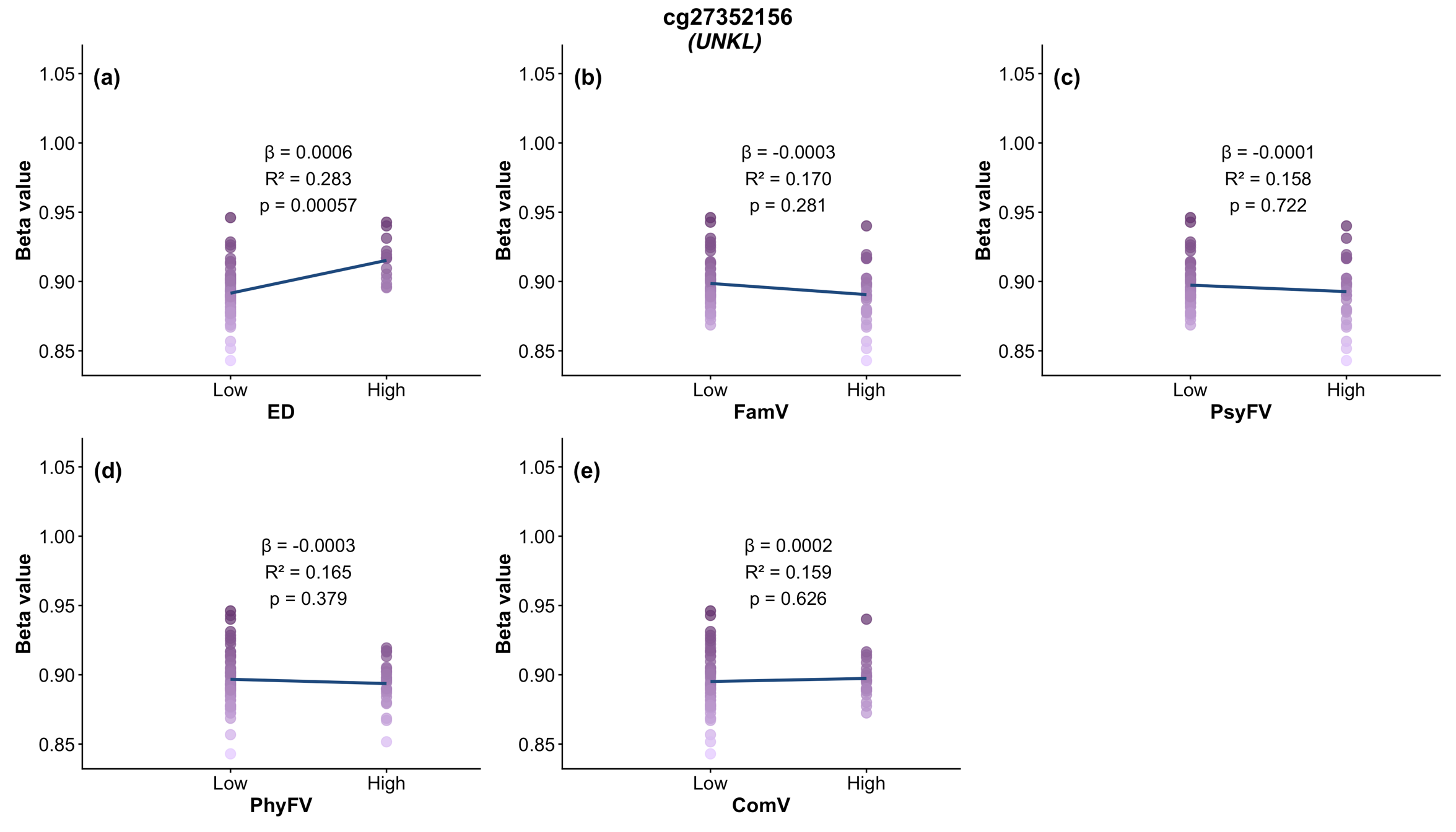
**

**Figure S5. Linear regression models of differential methylation (beta-values) at CpG site cg27352156 (*UNKL*) across (a) ED; (b) FamV; (c) PsyFV; (d) PhyFV; and (e) ComV.**


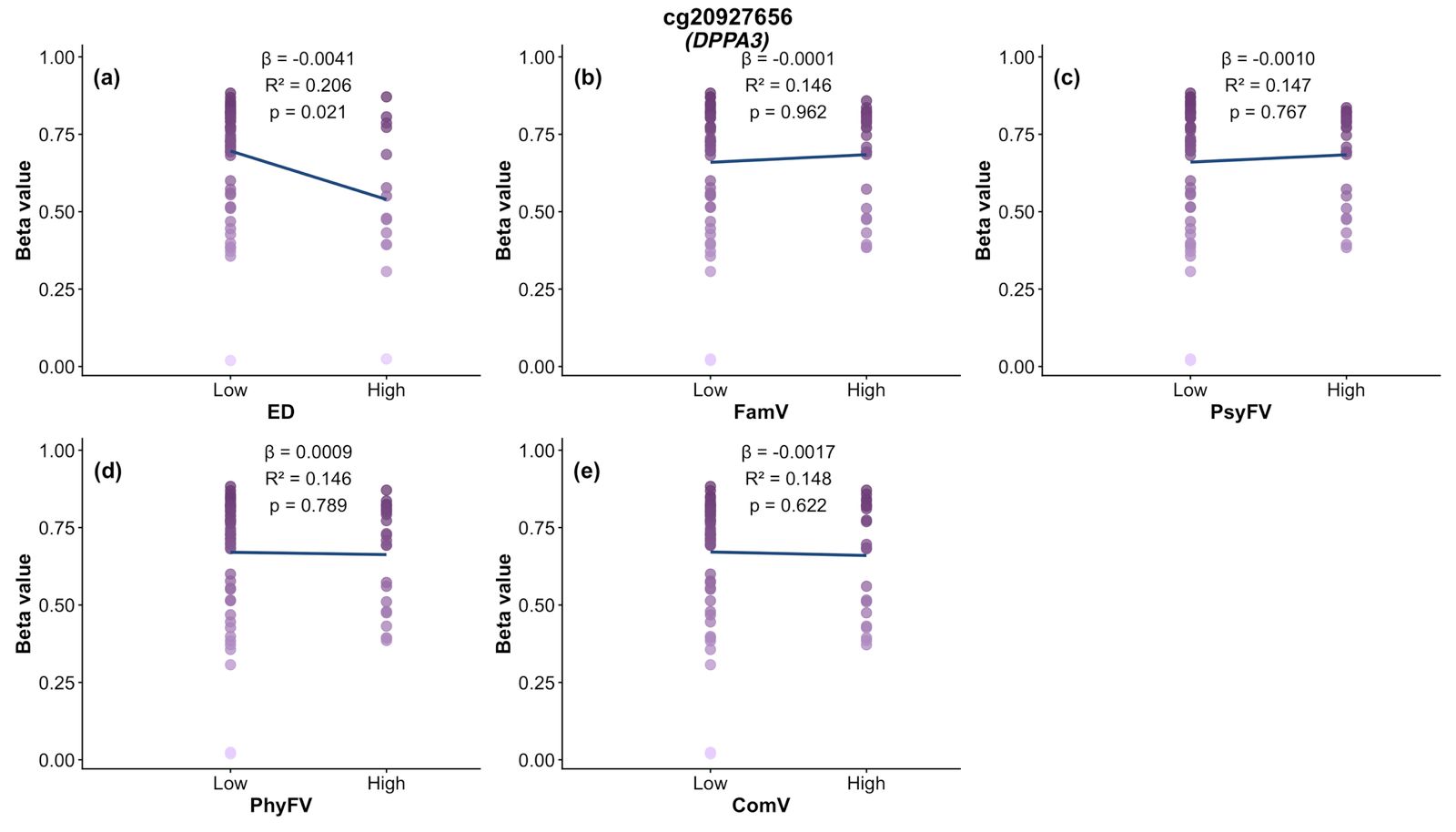


**Figure S6. Linear regression models of differential methylation (beta-values) at CpG site cg20927656 (*DPPA3*) across (a) ED; (b) FamV; (c) PsyFV; (d) PhyFV; and (e) ComV.**


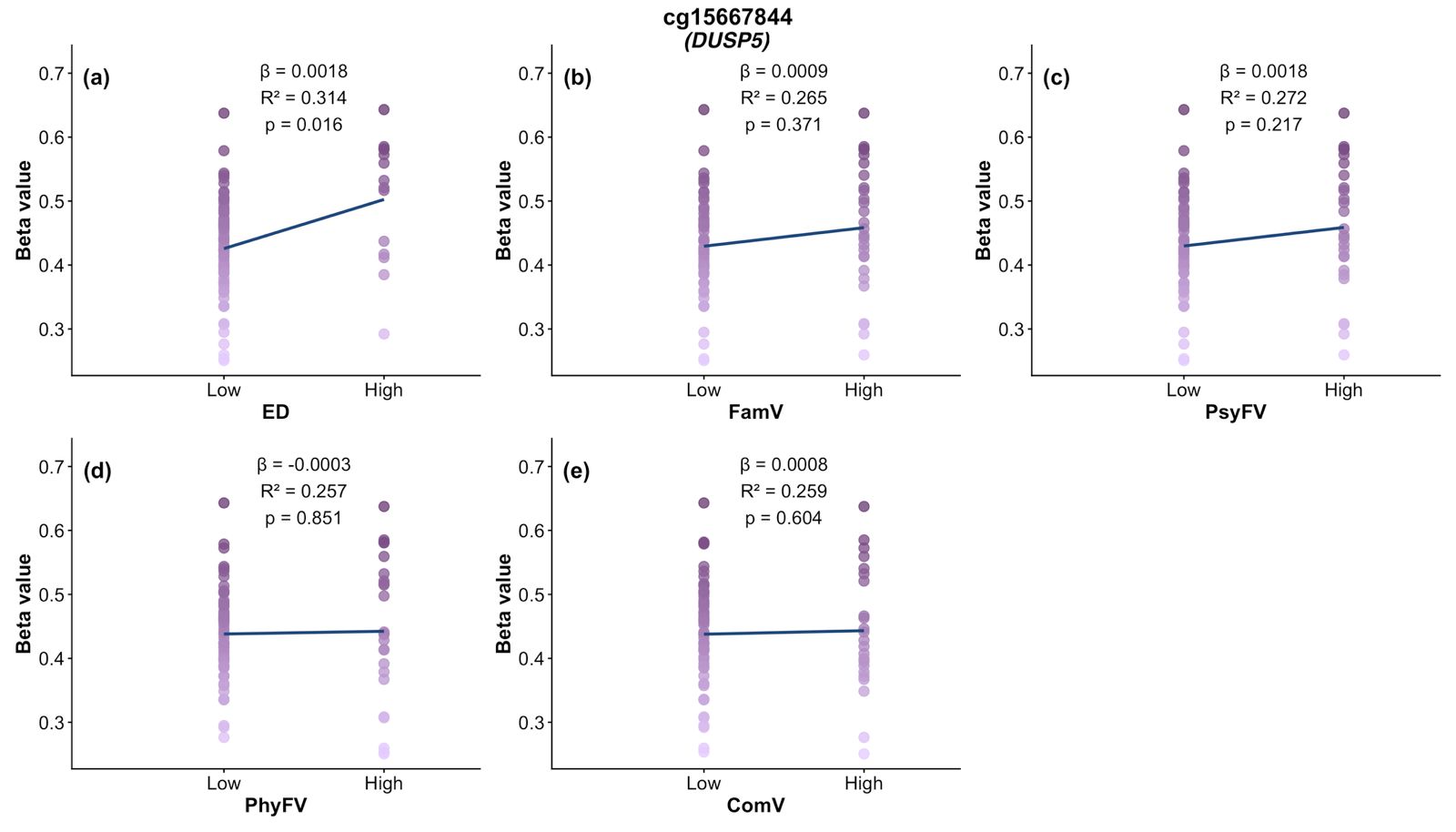


**Figure S7. Linear regression models of differential methylation (beta-values) at CpG site cg15667844 (*DUSP5*) across (a) ED; (b) FamV; (c) PsyFV; (d) PhyFV; and (e) ComV.**


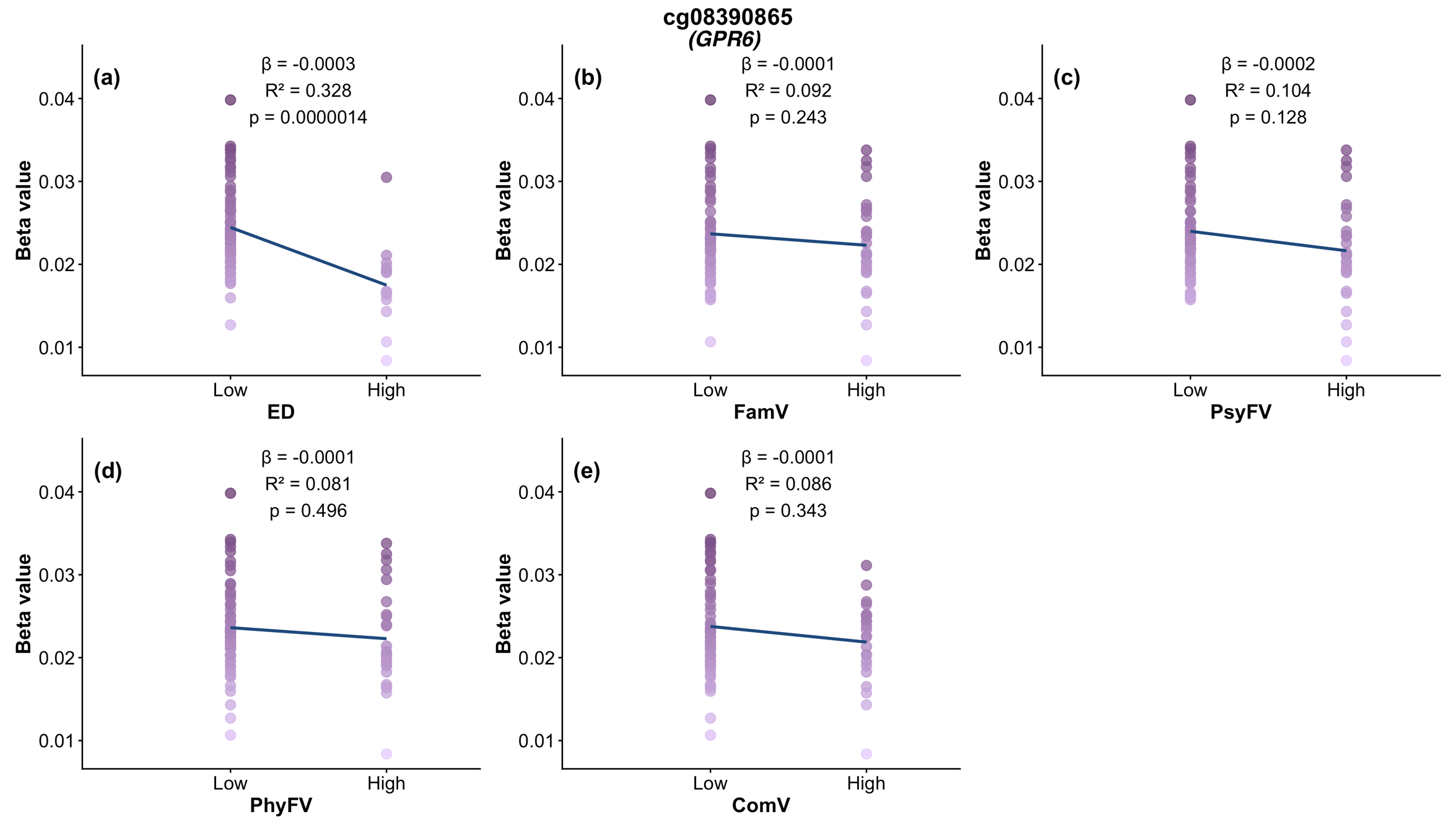


**Figure S8. Linear regression models of differential methylation (beta-values) at CpG site cg08390865 (*GPR6*) across (a) ED; (b) FamV; (c) PsyFV; (d) PhyFV; and (e) ComV.**


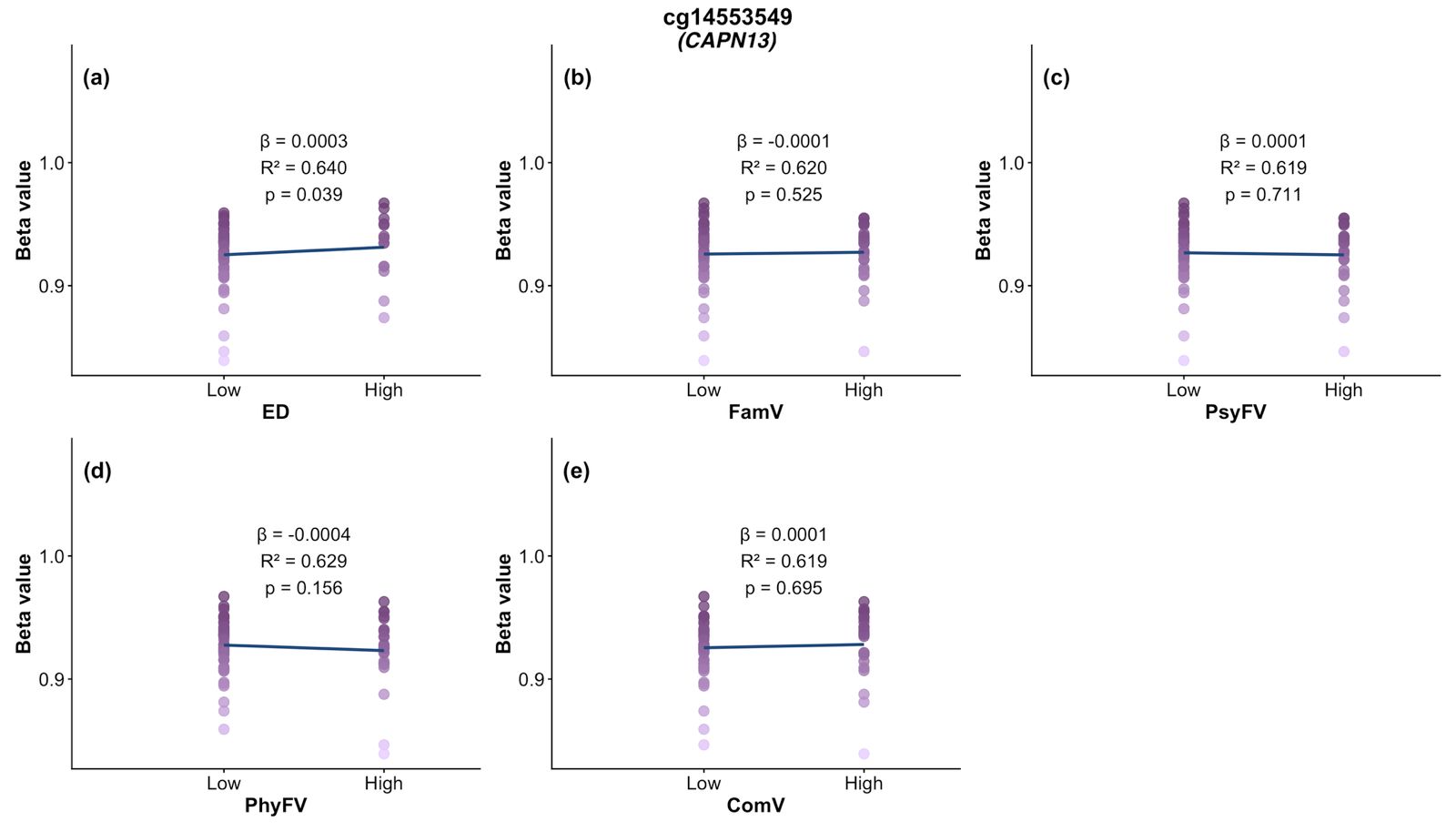


**Figure S9. Linear regression models of differential methylation (beta-values) at CpG site cg14553549 (*CAPN13*) across (a) ED; (b) FamV; (c) PsyFV; (d) PhyFV; and (e) ComV.**


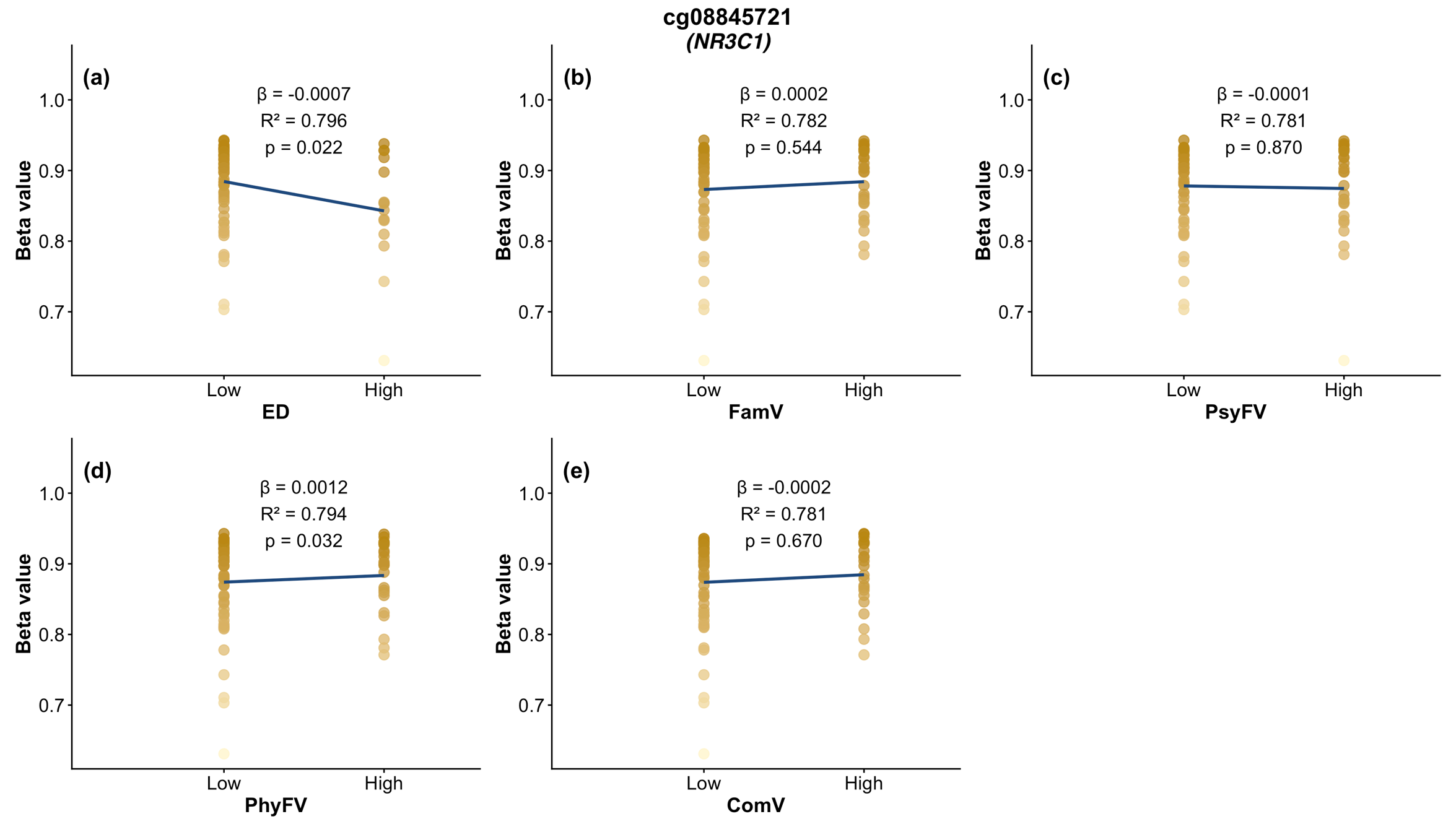


**Figure S10. Linear regression models of differential methylation (beta-values) at CpG site cg08845721 (*NR3C1*) across (a) ED; (b) FamV; (c) PsyFV; (d) PhyFV; and (e) ComV.**


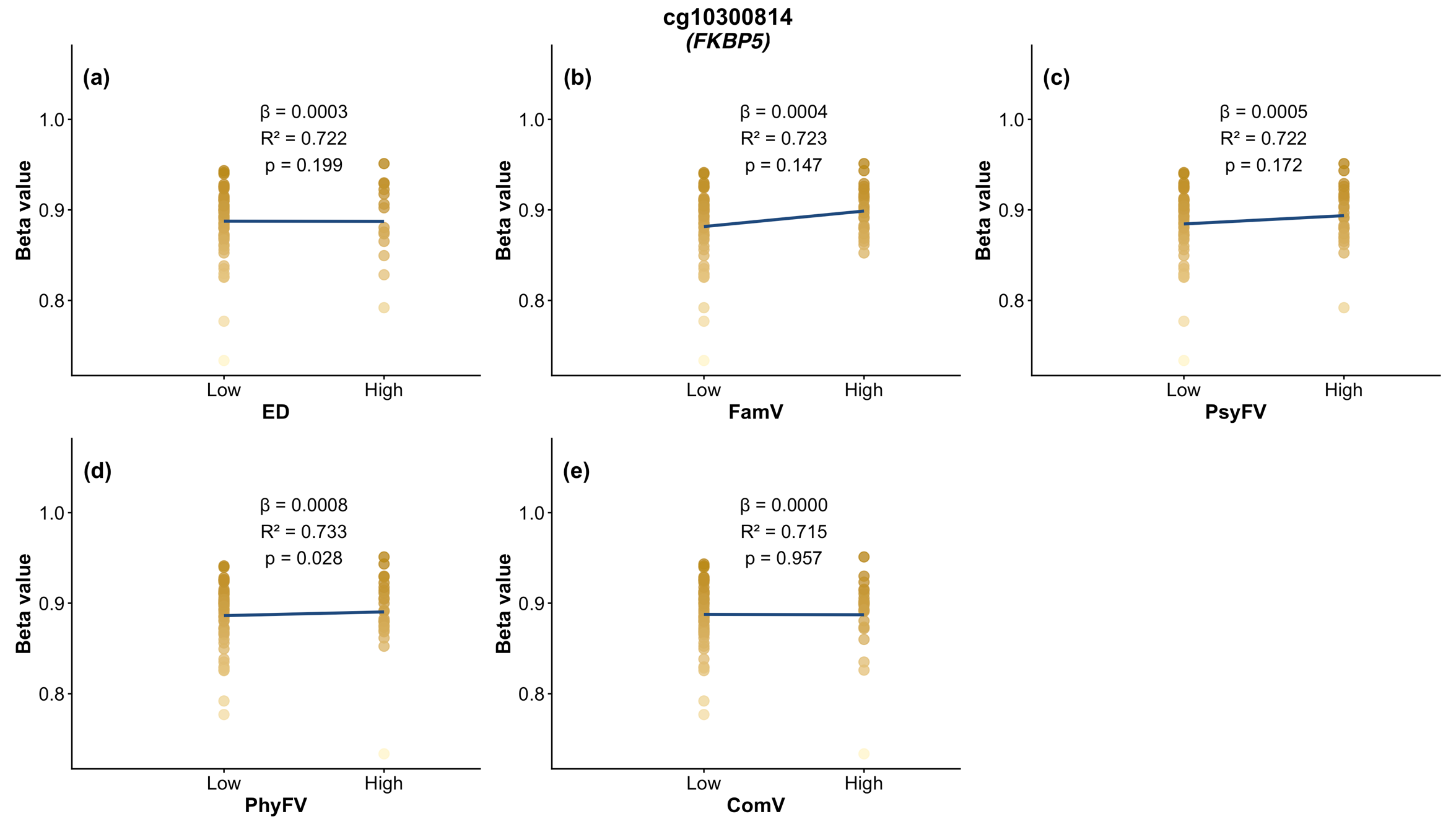


**Figure S11. Linear regression models of differential methylation (beta-values) at CpG site cg10300814 (*FKBP5*) across (a) ED; (b) FamV; (c) PsyFV; (d) PhyFV; and (e) ComV.**
